# Affect Dysregulation in Context: Implications and Future Directions of Experience Sampling Research on Affect Regulation Models of Loss of Control Eating

**DOI:** 10.3389/fpsyt.2021.747854

**Published:** 2021-09-27

**Authors:** Megan E. Mikhail

**Affiliations:** Department of Psychology, Michigan State University, East Lansing, MI, United States

**Keywords:** binge eating, loss of control eating, negative affect, emotion regulation, experience sampling, ecological momentary assessment

## Abstract

Loss of control eating is a core, transdiagnostic eating disorder symptom associated with psychological distress, functional impairment, and reduced quality of life. However, the factors that contribute to persistent loss of control eating despite negative consequences are not fully understood. Understanding the mechanisms that maintain loss of control eating is crucial to advance treatments that interrupt these processes. Affect regulation models of loss of control eating hypothesize that negative emotions trigger loss of control eating, and that loss of control eating is negatively reinforced because it temporarily decreases negative affect. Several variations on this basic affect regulation model have been proposed, including theories suggesting that negative affect decreases during loss of control eating rather than afterwards (escape theory), and that loss of control eating replaces one negative emotion with another that is less aversive (trade-off theory). Experience sampling designs that measure negative affect and eating behavior multiple times per day are optimally suited to examining the nuanced predictions of these affect regulation models in people's everyday lives. This paper critically reviews experience sampling studies examining associations between negative affect and loss of control eating, and discusses the implications for different affect regulation models of loss of control eating. The review concludes by proposing an expanded affect-focused model of loss of control eating that incorporates trait-level individual differences and momentary biological and environmental variables to guide future research. Clinical implications and recommendations are discussed.

## Introduction

Loss of control eating (LOC; i.e., eating accompanied by a sense of not being able to control what or how much one is consuming) is a transdiagnostic eating disorder (ED) symptom associated with considerable psychological distress (1), functional impairment (2), and reduced quality of life (3). LOC also plays a key role in perpetuating cycles of disordered eating. The transdiagnostic cognitive-behavioral model of EDs suggests that concerns about weight gain following LOC can lead to increased restriction, compensatory behaviors, and poor body image, which can in turn lead to more LOC in a maladaptive cycle (4). LOC and related disorders [e.g., bulimia nervosa (BN), binge-eating disorder (BED)] also represent a substantial public health concern. Approximately 10% of women, and a smaller but significant number of men, experience clinically significant binge eating (5). While there are empirically supported treatments for LOC, many who receive treatment continue to experience symptoms (6).

Though current DSM-5 diagnostic criteria focus on objective binge eating (OBEs; i.e., LOC over an objectively large amount of food), other forms of LOC and dysregulated eating that do not meet the formal definition of an “objective binge” in DSM-5 [e.g., subjective binge eating (SBEs), or LOC over relatively small amounts of food] may be even more prevalent (7) and associated with equivalent distress and comorbidity (8, 9). People with OBEs and people with SBEs are indistinguishable on numerous relevant variables, including dietary restraint, cognitive ED symptoms (e.g., body image concerns), disinhibition, general psychopathology, impairment, health service utilization, and endorsement of having an eating problem (9, 10). In contrast, overeating without LOC is associated with significantly less distress and impairment than OBEs and SBEs (11). Correspondingly, ICD-11 criteria treat OBEs and SBEs as equivalent, with either behavior sufficient to meet the binge eating criterion for BN and BED (12). Factor analytic studies also indicate that disordered eating is dimensional rather than categorical (e.g., OBEs and SBEs may represent different points on a dysregulated eating continuum) (13). In recognition of evidence regarding the dimensional nature of LOC, and evolving diagnostic standards that treat OBEs and SBEs as diagnostically equivalent, this review will examine a broad construct of LOC that includes OBEs, SBEs, and emotional eating (i.e., eating in response to negative emotions). Though emotional eating is not explicitly defined as involving loss of control, it shows robust empirical associations with other forms of LOC (14, 15), and similar patterns of change in response to negative affect (16) and biological fluctuations [e.g., ovarian hormone levels (17)], suggesting it is a closely related behavior. By incorporating multiple forms of LOC, this review will be poised to directly examine whether reproducible differences in the factors maintaining LOC over different amounts of food have been found.

Understanding the etiology and maintaining factors underlying LOC is crucial for advancing research and treatment. Several prominent etiologic theories highlight the role of negative affect (NA; i.e., negative emotions, including guilt, anger, and sadness) and emotion regulation difficulties in promoting LOC. These theories are consistent with evidence of elevated LOC among individuals with internalizing disorders (e.g., anxiety, depression) (18), and increased rates of internalizing disorders among people with EDs characterized by LOC (19). In other words, LOC may be one manifestation of more general negative emotionality and underlying difficulties with managing negative emotions among people with LOC-related disorders. This conceptualization has led to adaptation of treatments developed for other disorders characterized by affect dysregulation, such as dialectical behavior therapy (DBT), to BN and BED (20). Interestingly, treatments such as DBT that focus on more general emotion dysregulation appear to decrease comorbid psychopathology and other maladaptive behaviors (such as self-injury) in addition to LOC (21), suggesting that addressing affect dysregulation may be crucial to improving both core ED symptoms and accompanying concerns that contribute to distress/impairment.

**Figure 1:**
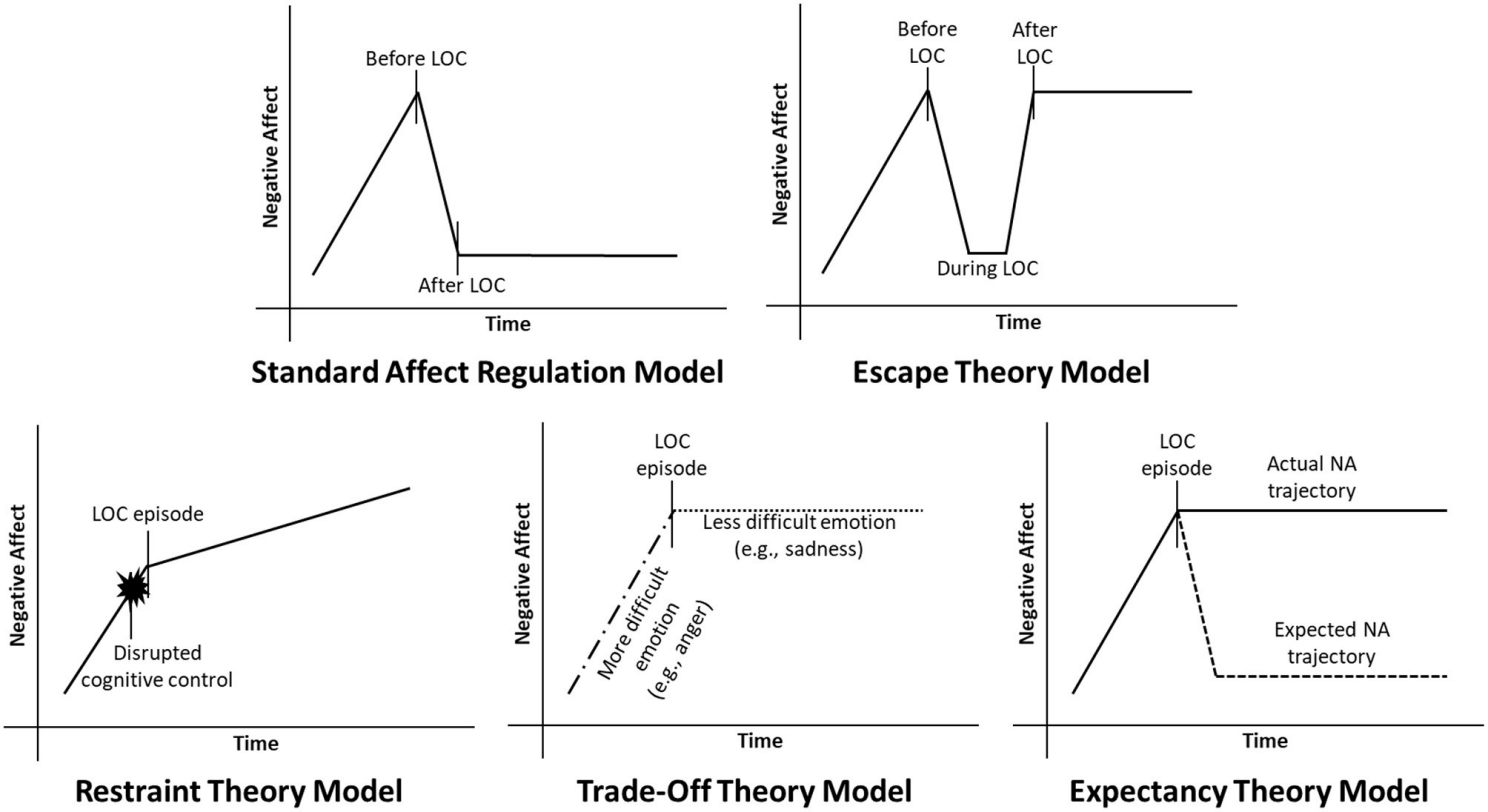
Current affect regulation models of negative affect (NA) and loss of control eating (LOC). In the standard affect regulation model, NA increases prior to LOC, and decreases following LOC. Conversely, escape theory predicts that NA decreases while LOC is in progress, but increases to pre-LOC levels after LOC. Restraint theory predicts that NA disrupts the cognitive control required to maintain dietary restraint, leading to LOC. Trade-off theory predicts that LOC replaces one negative emotion (e.g., anger) with another that is less aversive (e.g., sadness). Finally, expectancy theory predicts that people engage in LOC because they *expect* to feel better afterwards, even though they may not *actually* feel better.

Several affect regulation models have been developed, each of which proposes a somewhat different mechanism underlying the association between NA and LOC (see Figure 1). In its most straightforward iteration, the standard affect regulation model of LOC posits that LOC is triggered by an increase in NA, and is negatively reinforced by a decrease in NA following LOC (22). Operationally, this theory predicts that NA should be greatest just before LOC, and decrease following LOC. Related theories have proposed slightly different functions of LOC and trajectories of NA surrounding this behavior. For example, escape theory suggests that LOC serves to distract an individual not only from general NA, but specifically from negative awareness or attributions about the self (23). Like the standard affect regulation model, escape theory predicts that NA is elevated prior to LOC and triggers the onset of this behavior. However, escape theory differs from the standard affect regulation model in predicting that NA only decreases while the eating episode is ongoing, and increases again following LOC once self-awareness is reestablished.

Three additional theories diverge from the standard affect regulation model in hypothesizing that NA may trigger LOC, but LOC does not necessarily downregulate NA in turn. Restraint theory proposes that NA temporarily disrupts the cognitive control required to maintain food restriction, leading to dysregulated eating (24). In this case, NA could potentially continue to *increase* following LOC. Alternatively, trade-off theory posits that LOC may replace a highly aversive negative emotion (e.g., anger) with one that is more easily tolerated (e.g., sadness, guilt) (25). The overall intensity of NA may therefore remain unchanged following LOC, but the specific type of emotion experienced may shift. Finally, expectancy theory hypothesizes that *beliefs* regarding the affect regulatory properties of eating may drive LOC to a greater extent than the actual consequences of this behavior. Specifically, expectancy theory proposes that the expectation that one will feel better after eating increases the likelihood of eating to relieve NA, even if one's mood does not actually improve (26).

While all affect regulation theories predict that elevated NA may trigger LOC, they diverge in their predictions regarding trajectories of NA during and after LOC. The affective consequences of LOC are difficult to measure with the granularity necessary to distinguish between affect regulation theories using questionnaires administered once or at a limited number of timepoints, and may be distorted by the artificial and unfamiliar laboratory context in experimental research. Instead, changes in affect during and after LOC are optimally examined using experience sampling designs, which ask participants about their emotions and eating behavior in the context of their everyday lives. During experience sampling, questionnaires are administered once per day for several days (in daily diary studies) or multiple times per day [in ecological momentary assessment (EMA) studies], providing information about changes in emotions over relatively short timespans. Experience sampling designs have been used to study dynamic affective processes across EDs and other forms of psychopathology (27), and provide unique data regarding trajectories of affect in the minutes or hours before, during, and after LOC. Experience sampling designs also allow for examination of individual differences in momentary affect-LOC associations. For example, it is possible to test the hypothesis put forth by expectancy theory that individuals who more strongly believe that eating will reduce NA will in fact be more likely to eat in response to negative emotions.

This paper will comprehensively review evidence from experience sampling studies (including both daily diary and EMA designs) that have examined NA-LOC relationships to determine which affect regulation theory or theories are best supported by current research. The review will address three primary questions. First, what do the data say regarding changes in NA before and after LOC in naturalistic settings (i.e., does NA tend to increase, decrease, or remain stable in the minutes and hours before and after LOC)? Second, are certain negative emotions (e.g., guilt and anger) particularly likely to precede LOC, and do these differ from the negative emotions that are most likely to follow LOC (as predicted by trade-off theory)? Third, are there individual differences in momentary NA-LOC associations, and, if so, do they align with existing affect regulation theories (e.g., expectancy theory)?

Studies published prior to January 1, 2021 were considered for inclusion. Relevant studies were identified by searching PubMed and PsycINFO using the terms (“binge eating” or “emotional eating” or “loss of control eating” or “dysregulated eating”) and (“ecological momentary assessment” or “experience sampling” or “daily diary”). Additional studies were identified through manual search of the reference lists of prior meta-analyses [e.g., (22)]. Studies were included if they used an EMA or daily diary design to examine associations between NA and OBEs, SBEs, or emotional eating in adults 18 or older. Children and adolescents were not included in the present review due to evidence of developmental differences in the etiology (28) and phenomenology (29) of LOC and related disorders in youth.

## Research Examining Patterns of NA Surrounding LOC in Daily Life

Three primary experience sampling designs have been used to examine changes in affect surrounding LOC in daily life. Daily diary designs, in which participants report on their affect and eating behavior once at the end of the day, are the least intensive. While daily diary designs represent the lowest burden to participants, they also provide less fine-grained data than more intensive designs. Conversely, daily diary designs are better suited to detecting longer-term associations between NA and LOC (e.g., over a period of days or weeks) than briefer, more intensive studies. Intermediate in intensity are EMA designs that collect information on a participant's affect at several semi-randomly distributed times throughout the day, signaled by an alert on a participant's cellphone or a similar device (i.e., signal-contingent ratings). Some EMA studies also ask participants to immediately report when they experience LOC (i.e., event-contingent ratings). Similar to daily diary designs, these kinds of EMA studies can be used to examine whether NA is elevated near LOC episodes, but provide greater temporal precision (i.e., a window of a few hours rather than an entire day). The most intensive experience sampling designs ask participants to report on NA at multiple timepoints during LOC itself. These are the only designs that provide information about a participant's emotions while LOC is in progress. However, this method risks altering the natural consequences of LOC by directing participants to focus on their emotions during the behavior.

Results from each of these study designs will be discussed, then synthesized with respect to their implications for affect regulation models of LOC. Study details (e.g., sample size and demographics, operationalizations of NA and LOC) and key findings for each study are summarized in Appendix A in Supplementary Material, along with an assessment of study quality (described in Appendix B in Supplementary Material).

### Daily Diary Studies

Two studies have examined prospective (i.e., next day) associations between NA and LOC (30, 31). Though assessment periods differed in these studies (2 weeks vs. 45 days), findings were consistent in showing that NA was elevated on days characterized by binge eating (30) or high levels of emotional eating (31). However, NA failed to predict binge eating or emotional eating on the next day. Conversely, greater binge eating or emotional eating on a given day predicted greater NA on the next day in both studies. In other words, these studies suggest that LOC may increase NA over the medium-term (i.e., from 1 day to the next).

Five additional studies examined whether NA was greater on days with LOC, but did not include prospective analyses. NA was elevated on days with LOC in all five studies (32–36) across both clinical and community-based samples [though some effects did not reach statistical significance due to small sample size; e.g., (34)]. Interestingly, NA was elevated on days characterized by LOC even when controlling for dietary restraint (35) or in comparison to days characterized by restriction in women with AN (32), suggesting that LOC is associated with NA even after accounting for co-occurring disordered eating symptoms.

Together, daily diary studies indicate that NA may be a proximal trigger for LOC on a given day, but not across longer time spans (i.e., from 1 day to the next). These studies also suggest that dysregulated eating may result in increased NA in the medium-term. Results are not necessarily inconsistent with the standard affect regulation model that posits an immediate decrease in NA following LOC, but suggest that any emotional relief obtained through LOC is likely short-lived.

### Ecological Momentary Assessment Studies of the Hours Surrounding LOC

In total, 60 studies were identified that examined changes in NA in the hours surrounding LOC through moderate intensity EMA methods.

#### Studies Examining Patterns of NA on Days Charactered by LOC

Three studies examined whether certain patterns of NA across the day (e.g., increasing NA from morning to afternoon) were particularly likely to predict LOC on that day. While these studies sample affect at a higher rate than the daily diary studies discussed above, they are conceptually similar in seeking to understand how LOC may be related to NA across an entire day, rather than just immediately before or after LOC. All three studies (37–39) were consistent with daily diary research in showing that LOC was more common on days characterized by persistently high or increasing NA than on days when NA was low throughout the day. Persistently high NA or increasing NA across the day were associated with LOC transdiagnostically in women with BN (38), women with AN (39), and obese adults with no history of AN or BN (37).

It is less clear how the amount of food consumed during LOC may affect associations between daily patterns of NA and eating behavior. Lavender et al. (39) only examined OBEs, and Crosby et al. (38) combined SBEs and OBEs in their assessment and analyses, making it difficult to determine whether effects were driven primarily by LOC episodes that were objectively large. When Berg et al. (37) examined SBEs specifically, they found that these episodes were no more likely on days with persistently high or increasing NA than on days with consistently low NA. In contrast, overeating without LOC was more likely on days with stable high or increasing NA. These results could suggest that high NA across the day is more closely associated with overeating than with LOC *per se*. However, disambiguation of LOC and overeating may have been challenging in Berg et al. (37) given that this study assessed a non-clinical population, and overeating was defined somewhat subjectively as a sense of having overeaten, rather than consuming an amount of food that others would consider objectively large. It is also striking that 96% of participants in Berg et al. (37) endorsed OBEs during the 2 weeks of the study, but only 24% met criteria for BED or subthreshold BED assessed through clinical interview at baseline. This discrepancy could perhaps suggest that participants' definitions of OBEs did not fully align with clinically significant OBEs as defined by DSM-5 (e.g., some may have in fact been SBEs). Additional research, including research in clinical populations, is therefore needed to determine whether associations between LOC and patterns of NA across the day differ based on the amount of food consumed.

#### Studies Examining Concurrent Associations Between NA and LOC

Three EMA studies used designs that made it difficult to disambiguate whether NA was an antecedent or consequence of LOC (e.g., designs that asked about affect and LOC concurrently). These studies all showed greater NA at the time of perceived binge eating (40, 41) or emotional eating (42).

#### Studies Examining NA as a Predictor of LOC Only

An additional 20 studies examined whether NA prospectively predicted LOC within a given day, but did not examine changes in NA following LOC. Nearly all studies found that greater NA predicted greater likelihood of LOC at the next assessment, at least under certain conditions. Positive associations between NA and subsequent LOC or binge eating urges have been found for individuals with BN or BED (43–50), women with AN (51), undergraduate and community participants with no threshold ED (52–55), and individuals presenting for weight loss treatment (56). These results suggest that NA may be an important antecedent of LOC across populations.

In addition to suggesting that NA tends to be elevated over a person's average before LOC, most research also indicates that NA is greater prior to LOC than prior to meals and snacks not characterized by LOC. For example, Davis et al. (57) found that women with BN reported greater NA in the hour before self-defined binge eating than in the hour before meals/snacks. Similarly, Engelberg and colleagues (58) found that greater NA prior to eating was associated with greater odds that an eating episode was an OBE, rather than a meal/snack, in women with threshold or subthreshold BN. NA also appears to be higher prior to LOC than other kinds of eating for individuals with BED and those who do not meet criteria for an ED (59), suggesting that elevated NA is a specific predictor of LOC (rather than eating in general) across diagnoses.

One exception to this general pattern is Sanftner and Crowther (60), which found that the increase in NA prior to self-defined binge eating in a sample of undergraduate women with OBEs but no compensatory behaviors was similar to that experienced prior to normal eating in a control group. They found that although women with OBEs tended to experience greater guilt and shame than women without OBEs at a trait level, this group difference did not increase prior to LOC episodes. Of note, while many of the above studies asked about NA immediately before eating, Sanftner and Crowther (60) examined affect 1–9 h before binge eating. Thus, it may be that NA rises rapidly in the hour before LOC rather than in a gradual fashion across the day (a possibility that is supported by studies examining the trajectory of NA around LOC episodes in greater detail; see below).

#### Studies Examining LOC as a Predictor of NA Only

Two studies examined whether NA was elevated after LOC, but did not include an analysis of pre-LOC affect (61, 62). These studies should be interpreted with caution because (as discussed above) NA tends to be elevated prior to LOC. Thus, without a pre-eating affect rating, it is hard to distinguish NA resulting from LOC from NA carried over from the minutes prior to LOC. Both studies found elevations in NA following LOC, though this was qualified somewhat by individual differences in impulsivity in Smith et al. (61).

#### Studies Examining Absolute Changes in NA From Before LOC to After LOC

Nineteen studies used a pre/post design to examine absolute changes in NA from EMA assessments completed before LOC to EMA assessments completed after LOC. These studies used one of two sampling approaches. In the first approach (used by 12 studies), NA was assessed at multiple times throughout the day, and NA at the last signal prior to a reported LOC episode was compared to NA at the next signal following LOC. One benefit of this approach is that affect is captured around eating episodes even if the participant did not know LOC would occur when they started eating. This approach may also be less susceptible to reactivity [i.e., a change in behavior that results from being observed (63)], as LOC may not occur until several minutes or even hours after the last affect rating was recorded. However, a shortcoming is that pre- and post-eating ratings may not be equally temporally spaced around the eating episode (e.g., a pre-eating rating could have been made an hour before an LOC episode, and the post-eating rating only minutes after LOC).

Results from studies using this approach were relatively consistent in showing that NA was greater after LOC than before LOC. This result was replicated in eight out of 12 studies, including studies of women with AN (64, 65), women with BN (64, 66–68), women with BED (69), adults with type I diabetes (70), and undergraduate women with no threshold ED diagnosis (71). One additional study found that negative emotions were elevated both before and after LOC relative to other eating episodes, but could not directly compare pre- to post-eating ratings because different measures were used at pre/post timepoints (72). The frequency of assessment in these studies varied from more than once per hour (70) to once every 2.5–3 h (71), suggesting a relatively persistent increase in NA post-LOC that remains detectable hours after the behavior. While most studies examined OBEs only, Engel et al. (65) found an increase in NA after any form of LOC (including SBEs or OBEs).

The three studies that yielded different results have interesting potential implications for our understanding of NA-LOC associations. Of all 12 studies, only Lavender et al. (73) found an apparent *negative* association between LOC and later NA in women with BN. However, once methodological differences are accounted for, results of Lavender et al. (73) are more similar to the above studies than they initially appear. While LOC was negatively associated with NA (as well as purging) at the next EMA signal, it was *positively* associated with NA measured immediately after LOC. In other words, LOC may be associated with an immediate increase in NA, followed by a decrease in NA from this peak that is greater than would typically be observed over the same time period from a neutral point in the day (rather than an absolute decrease in NA).

Similar to Lavender et al. (73), Wegner et al. (74) found a more complex pattern of associations between LOC and NA over time in undergraduate women with recurrent OBEs. Mean levels of NA averaged across the day were significantly higher on days characterized by LOC. In contrast, NA at the first signal after LOC did not significantly differ from NA at the last signal prior to LOC (approximately 2 h apart). However, immediately following LOC, participants reported significantly worse moods than they recalled experiencing before LOC. NA reported immediately after LOC was also notably higher than the average level of NA on LOC days. These findings suggest that NA may be elevated immediately after LOC, but that this increase may be relatively brief for individuals who don't meet full criteria for a threshold ED. Intriguingly, results also indicate that people with LOC themselves perceive LOC as worsening their mood, at least immediately after the behavior. Correspondingly, when Stein et al. (69) asked women with BED why they thought they experienced LOC during EMA, participants attributed the onset of nearly half (47.7%) of binges to how they felt, but only rarely reported stopping eating because of a change in their emotions (5.5%).

Svaldi et al. (75) also found that NA was greater on days with OBEs in women and men with BED, but did not find a significant difference between NA in the hours before and after OBEs. Notably, Svaldi et al. (75) only sampled affect three times throughout the day, and prompts could be as far as 8 h apart. Therefore, this study may have missed short-lived increases in NA occurring immediately after OBEs. Because most other studies have only included women, a second potential explanation is that the affective consequences of LOC may differ across sex. Smaller (or no) increases in NA following LOC in men could have potentially weakened the association in the overall sample. Unfortunately, Svaldi et al. (75) did not conduct moderation analyses across sex to examine this possibility.

A second approach to examining absolute changes in affect across LOC (used by seven studies) is to ask participants to rate their affect immediately before and immediately after LOC. This approach has the advantage of capturing the most proximal affective antecedents and consequences of LOC. However, it is possible that drawing a person's attention to their emotional state right before or after LOC could alter the effects of eating on their emotions (e.g., a person may become more focused than they otherwise would on guilt about gaining weight).

All studies in this category showed increases in NA following LOC, but with some nuances with respect to who experienced these increases, and when. Alpers and Tuschen-Caffier (76) and Sherwood et al. (77) both found an increase in NA immediately following LOC in women with threshold BN. Findings were similar in women with subthreshold BN in Sherwood et al. (77). Sherwood et al. (77) also found that NA continued to increase in the hour following LOC in women with both threshold and subthreshold BN, and that NA did not significantly change after normal meals in either group. Elmore and de Castro (78) likewise observed that women with current BN experienced an increase in feelings of depression following LOC, but not following normal meals. However, when parsing LOC episodes further, Elmore and de Castro (78) found that feelings of depression only increased significantly from pre- to post-LOC for LOC episodes that were followed by purging. This could indicate that particularly high levels of NA following LOC may be a trigger for purging in women with BN.

Goldschmidt et al. (79) dismantled components of eating episodes to examine whether an increase in NA post-eating was primarily a consequence of LOC, eating an objectively large amount of food, or their combination. Both OBEs and SBEs were associated with greater pre-eating and post-eating NA than occurrences of normal eating. By contrast, normal eating and overeating without perceived LOC did not differ on pre- or post-eating NA. Additionally, NA increased pre- to post-eating for OBEs and SBEs, but decreased across overeating and normal eating episodes. Findings suggest that LOC, rather than perceived overeating, may be the “active ingredient” driving proximal increases in NA. Nevertheless, when OBEs and SBEs were directly compared in Goldschmidt et al. (79), OBEs were associated with greater pre- and post-eating NA than SBEs. Among LOC episodes, therefore, greater initial NA may drive consumption of a greater number of calories, which may in turn contribute to a greater increase in NA for OBEs than SBEs.

In summary, studies that measured NA shortly before and after LOC consistently suggest that NA is greater immediately following LOC. NA may then decrease in the hours following LOC, but appears to remain elevated over NA on days without LOC. As observed by Haedt-Matt and Keel (22) in an earlier meta-analysis of changes in NA following LOC, these results do not neatly align with the standard affect regulation model of LOC positing that LOC is reinforced by a reduction in NA.

#### Studies Examining Trajectories of NA Before and After LOC

Seventeen studies examined trajectories of NA before and after LOC. Rather than focusing primarily on whether the absolute level of NA was higher or lower pre- to post-LOC (as in the studies discussed above), these studies investigated whether NA was increasing or decreasing (i.e., the slope of change in NA over time was positive or negative) before and after LOC. In contrast to research examining absolute levels of NA before and after LOC, 15 out of 17 studies found that NA was on an increasing trajectory prior to LOC, but a *decreasing* trajectory following LOC. Decreasing NA in the hours following LOC has been found in women with threshold or subthreshold BN (64, 80–87), women with comorbid BN and borderline personality disorder (88), women with threshold or subthreshold AN (64, 65, 81, 89), men and women with BED (90, 91), and obese adults with no history of AN or BN (92).

Only two studies failed to find significant changes in the trajectory of NA after LOC (75, 93). As discussed above in the section on absolute changes in NA before and after LOC, Svaldi et al. (75) may have been less able to detect changes in the trajectory of NA after LOC due to relatively infrequent sampling. Close inspection of the figures in Stevenson et al. (93) shows that most forms of NA were in fact decreasing after LOC, but not strongly enough to reach statistical significance with a relatively small sample (*N* = 48). Effects may have also been more subtle in Stevenson et al. (93) due to analysis of all LOC episodes, rather than OBEs alone. While most studies examining the slope of NA following LOC analyzed OBEs only or combined OBEs and SBEs in analyses, Berg et al. (92) explicitly investigated whether there were differences in the trajectories of NA surrounding OBEs and SBEs among obese adults with no history of AN or BN. The trajectory of NA was significant and negative after OBEs in Berg et al. (92), but was not significantly different from zero following SBEs. The estimated level of NA at the time of eating was also greater for OBEs than for SBEs. While replication is needed, these results could suggest that a decreasing trajectory of NA is more likely following OBEs.

When considering studies examining trajectories of NA surrounding LOC together with the pre-/post-LOC studies described above, an apparent contradiction in findings emerges. How can it be that NA is greater after LOC than before LOC, but also decreases following LOC? This discrepancy across analytic methods has been replicated in the small number of studies that examined both absolute changes in NA and changes in the trajectory of NA following LOC in the same sample (64, 65), suggesting that the apparent inconsistency likely cannot be attributed solely to differences in study methodology or participants.

Berg et al. (64) suggested that disparate findings across analytic methods may be an unintended consequence of the timing of EMA assessments. Specifically, Berg et al. (64) found that, on average, LOC episodes tended to be closer in time to the first assessment immediately following the episode (approximately 15–20 min later) than to the last assessment immediately prior to the episode (over 2 h before). Berg et al. (64) hypothesized that this pattern could result from use of event-contingent recordings that ask participants to report their mood immediately after LOC, without a similar assessment automatically initiated prior to LOC.

While the rationale provided by Berg et al. (64) may help explain the absolute increase in NA following LOC among studies that examined affect at randomly timed signals throughout the day, it cannot account for the results of studies that required participants to report their affect both immediately before and immediately after LOC [e.g., (76, 77, 94)]. The fact that NA has fairly consistently been found to be greater post-LOC than pre-LOC in studies assessing affect immediately before and after eating suggests that NA may in fact increase immediately after LOC.

In exploring what else may account for discrepant findings between pre/post and trajectory analytic strategies, it is worth noting that the decrease in NA following LOC in studies that used the trajectory method may not be as steep as the increase in NA prior to LOC. This was the case for Berg et al. (64), which predicted a higher level of NA post-LOC than pre-LOC at equivalent removes from the LOC episode (e.g., an hour before LOC vs. an hour after LOC), even though NA was found to be decreasing post-LOC. Similarly, Engel et al. (65) found a curvilinear increase in NA prior to LOC, followed by a considerably more gradual, linear decrease in NA following LOC. Munsch et al. (90) likewise found that NA was greater 30 min after OBEs than 30 min before OBEs, even though NA appeared to be on a decreasing trajectory following OBEs. These studies suggest that NA may be greater shortly after LOC than before LOC, even if it begins to decrease in subsequent hours.

Studies of the trajectory of NA surrounding LOC also largely leave unaddressed the timing of the peak in NA, and particularly whether this peak occurs before, during, or after LOC. This point is conceptually important because delayed reinforcement (for example, a gradual decrease in NA following an initial increase after LOC) may be less effective at shaping behavior than immediate reinforcement (95), and could suggest other primary maintaining factors for LOC. Munsch et al. (90) and Smyth et al. (83) indirectly addressed this issue by excluding mood assessments that occurred near the LOC episode (within 30 or 10 min, respectively) and could be attributable to the episode itself. Results of these analyses were somewhat mixed. When assessments immediately surrounding LOC episodes were excluded, Munsch et al. (90) found that NA appeared to be decreasing both before and after LOC episodes, while Smyth et al. (83) continued to find that NA was increasing before LOC and decreasing after LOC. These results suggest that there may be relatively little time (perhaps no more than about 30 min) on average between when NA begins to rise and when LOC occurs, and that NA may begin to decline soon after LOC (possibly within 10 min). However, they do not rule out a peak in NA immediately after LOC, or the possibility that NA may remain elevated above baseline for some time later.

Additionally, studies examining the trajectory of NA after LOC have not typically investigated whether LOC is associated with a steeper decrease in NA than would be observed over a similar timespan if a person did not engage in LOC. One exception is Stevenson et al. (93), which found that the trajectory of NA following LOC did not significantly differ from the trajectory of NA at the same time of day on days without LOC (abet, as mentioned above, in a relatively small sample). An even more robust comparison would be to examine differences in the trajectory of NA after unpleasant events (e.g., difficult interpersonal interactions) or equivalently intense NA ratings that were and were not followed by LOC. This is because we would statistically expect a decrease in NA following an unusually high point [i.e., “reversion to the mean” (96)], even if LOC itself had no impact on the trajectory of NA. If LOC does not dramatically hasten the decline in NA following a peak, this may be a useful insight for individuals who believe that LOC causes them to feel better. Indeed, a similar insight has been used in treatment of anxiety disorders to discourage maladaptive behaviors that prevent individuals from learning that negative emotions tend to decrease over time on their own [e.g., (97)].

Altogether, the data suggest that NA decreases in the hours following LOC, but whether this decrease is caused by LOC remains unclear. Research across analytic methods also indicates that NA remains higher post-LOC than pre-LOC. Nevertheless, it is possible that individuals who experience LOC may come to believe that this behavior is responsible for the eventual decline in NA that follows a peak in uncomfortable emotions, and eat in response to their emotions to achieve this desired consequence. It is also possible that other perceived benefits of LOC may outweigh the consequences of a temporary increase in NA. For example, LOC is often proceeded by food restriction in daily life (98). Temporary “permission” to eat freely and consume otherwise forbidden (often highly palatable) foods, which may be intrinsically pleasurable (99), could in some cases be perceived as worth the cost of a temporary elevation in NA.

### Ecological Momentary Assessment Studies Assessing Affect During LOC

Five studies assessed affect over the course of LOC episodes themselves. These studies can provide unique insight into an individual's affective experience as they are engaging in LOC. However, the intensive monitoring that is the inherent strength of these studies may also risk altering the experience and consequences of LOC.

Results of studies examining NA across LOC episodes are mixed, with some suggesting a decrease in NA during LOC (consistent with escape theory) (100, 101), and others indicating unchanged or increased NA during LOC (102, 103). Notably, and consistent with the pre-/post-LOC designs described above, all studies found that NA increased after LOC to levels similar to, or greater than, before LOC (100–104). Use of compensatory behaviors after OBEs was associated with a decrease in NA in two studies that examined women with BN (102, 104). However, NA in these studies was no lower after use of compensatory behaviors than before OBEs, and some negative emotions (particularly guilt/shame) remained elevated over pre-LOC levels even after purging. These results add to evidence suggesting that LOC is unlikely to result in lasting relief from NA, even when accompanied by compensatory behaviors to counteract potential effects on body weight/shape.

Reasons for discrepant findings regarding NA during LOC are not immediately evident. The two studies (100, 101) that most clearly observed a decrease in NA during LOC examined undergraduates with OBEs but no compensatory behaviors, perhaps suggesting a difference in affective patterns based on the presence of compensation. When directly comparing women with diagnosed BN and BED, however, Hilbert et al. (103) observed a similar pattern of affect across OBEs in both groups, with NA remaining stable from before to during OBEs, then increasing after OBEs.

A second possible explanation is differences in the methods used to measure affect across studies. Most of the studies that did not find a decrease in NA during LOC asked participants to rate the extent to which they felt various emotions (e.g., anxiety, anger) on a Likert scale. In contrast, Deaver et al. (100) required participants to visually mark their affect on a grid representing dimensions of pleasantness and arousal, while Stickney et al. (101) included both a checklist of emotions and open-ended questions regarding participants' internal experiences. Both these methods arguably required a greater level of cognitive processing by participants than a simple Likert scale, either because they were unfamiliar [in the case of Deaver et al. (100)] or because they explicitly called for in-depth reflection in the moment [in the case of Stickney et al. (101)]. These more involved methods may have disrupted the natural affective course of LOC to a greater extent than less complicated emotion scales. That greater reflection would result in greater reductions in NA is somewhat counterintuitive given the predictions of escape theory. However, more intensive reflection about one's thoughts and emotions could potentially distract from the present moment experience and instead activate cognitions related to the expectation that LOC will improve mood. In other words, it is possible that participants who are asked to engage in intensive cognitive processing during LOC may report feeling better because they think they should, even if their mood typically would not improve during LOC.

### Patterns of Change Before and After LOC for Specific Negative Emotions

While most studies have considered NA as a composite construct (i.e., an overall sense of feeling “bad”), some have attempted to tease apart whether patterns of change for specific negative emotions differ across LOC (e.g., whether sadness follows a different trajectory than fear). One caveat to these analyses is that NA scales tend to show high internal consistency (105), suggesting that specific negative emotions may not be experienced as fully distinct. This may be particularly true for individuals with LOC, who may differentiate less between their emotions than people without a history of LOC (106). Nevertheless, identifying negative emotions that track more strongly with LOC may provide insight into the events and cognitive antecedents (e.g., critical thoughts about oneself vs. critical thoughts about others) that are most likely to trigger LOC.

Five studies examined changes in specific negative emotions from immediately before to immediately after LOC (69, 74, 78, 102, 107). As might be expected from the general high internal consistency of NA measures, most negative emotions, including guilt and depression/sadness, followed a similar pattern in these studies. Sadness, guilt, and other negative emotions directed at the self (e.g., anger at self) were greater on days with LOC than days without LOC, and greater immediately after LOC than immediately before LOC (69, 74, 78, 107). Anxiety was the only negative emotion that showed a somewhat less consistent pattern. In Elmore and de Castro (78) and Redlin et al. (107), anxiety decreased following binge eating. However, Powell and Thelen (102) found similar patterns of anxiety, depression, and hostility across the binge eating/purging cycle. Specifically, each negative emotion was experienced to a greater extent during and after OBEs than before OBEs, but decreased somewhat after use of compensatory behaviors.

Two additional studies that used intensive EMA designs to sample affect during LOC itself suggest there may be subtle differences between different negative emotions during LOC. Stickney and colleagues (101) found that worry, sadness, and irritability were slightly lower during LOC than before LOC, but guilt and frustration were the same or greater during LOC as before LOC. Nearly all negative emotions were greater after LOC than before LOC in Stickney et al. (101), but the increase in guilt and anger at self were particularly pronounced. Similarly, Corstorphine et al. (104) observed an increase in guilt following OBEs in women with BN that persisted even after vomiting. Thus, emotions reflecting a negative evaluation of oneself and one's behavior (guilt, anger at self) seem particularly likely to increase and stay elevated as a consequence of LOC.

Studies examining the trajectories of individual negative emotions surrounding LOC also suggest that guilt may be uniquely important. Two studies found that guilt was increasing prior to LOC and decreasing following LOC (similar to the trajectories of composite NA scores described above), but that the slopes of fear, hostility, and sadness were not significantly different from zero before or after LOC (91, 92). In women with BN, Berg et al. (85) found that fear, hostility, guilt, and sadness all increased prior to LOC and decreased following LOC, but only the changes in guilt remained statistically significant after controlling for the effects of other negative emotions. When applying the more stringent test of comparing trajectories of affect on days when LOC did and did not occur, Stevenson et al. (93) observed that the increase in guilt up to the estimated time of LOC was significantly greater than the increase in guilt over the same time period on days in which LOC did not occur. Conversely, the slope of guilt following the time of LOC was not significantly different on LOC and non-LOC days. In other words, guilt appeared to increase dramatically up to (and perhaps immediately after) LOC, and remained high following LOC. While anger, anxiety, sadness, and stress were all higher on days characterized by LOC than days without LOC in Stevenson et al. (93), the trajectories of these other negative emotions did not significantly differ between LOC and non-LOC days.

Rather than examining discrete negative emotions, Becker et al. (80) took a slightly different approach in grouping negative emotions based on their associated level of arousal and hypothesized avoidance/approach behavioral drives. All categories of negative emotions showed relatively similar trajectories surrounding LOC. However, participants reported experiencing more intense high arousal, avoidant negative emotions at the point of LOC than high arousal, approach negative emotions, suggesting that the experience surrounding LOC was primarily one of wanting to withdraw. Interestingly, low arousal, avoidant emotions showed the steepest decline following LOC only (but not LOC and purging) events. This finding is consistent with the observation by Stickney et al. (101) that boredom (a low arousal negative emotion) was the only negative emotion to notably decrease from immediately before to immediately after LOC. In a twist on trade-off theory, results suggest that LOC may in some cases replace low arousal, negative emotions (e.g., boredom) with high arousal, negative emotions reflecting an unfavorable self-appraisal (e.g., guilt/shame) that may in some ways be even more aversive.

### Patterns of NA Across Individual Differences and Within-Person Variables

Some people may be more generally prone to LOC after an increase in NA due to individual differences in personality (e.g., impulsivity), beliefs (e.g., expectations that eating will relieve NA), or diagnostic status. Additionally, how a person feels at a given moment may affect the association between NA and LOC. Three potential moderators of NA-LOC associations are discussed below: diagnostic categories, eating expectancies (i.e., beliefs about the impact of eating on emotions), and negative urgency/impulsivity. These factors were chosen for their relevance to affect regulation models and because they have been examined in multiple studies, and thus have the greatest body of evidence from which to draw conclusions.

#### Diagnostic Categories

As indicated in the sections above, the overall pattern of NA surrounding LOC appears relatively similar across diagnoses. LOC tends to be preceded by increased NA in both people with (43–45, 48–50, 94) and without (52–54, 79) a diagnosable ED. Immediate increases in NA following LOC also appear similar in individuals with threshold and subthreshold EDs [e.g., women with threshold and subthreshold BN (77)], as do eventual declines in NA in the hours following LOC (64, 65, 80–85, 90, 92). However, some studies do suggest that associations between NA and LOC may be *stronger* when LOC is experienced over larger amounts of food (37, 79, 92), or by individuals whose disordered eating is severe and persistent enough to meet the threshold for a diagnosis (108, 109) or cause significant distress (62). In the only study to examine the impact of neurobiological differences on NA-LOC associations, Steiger et al. (94) also found that increases in NA following LOC were greater in women who displayed more pronounced irregularities in serotonin function. Differences between individuals with threshold and subthreshold diagnoses could therefore reflect different degrees of altered functioning in neural circuits important for mood and emotion processing. Alternatively, or additionally, it could be that larger and more frequent LOC episodes are associated with greater subsequent emotional distress because of their greater cumulative potential impact on body weight and shape.

#### Eating Expectancies

Expectancy theory predicts that LOC will be particularly likely to follow an increase in NA among people who believe that eating will improve how they feel. Several studies have shown that LOC is more common among people who believe that eating will relieve negative emotions [e.g., (48, 53)]. However, only one study has directly tested the hypothesis that NA-LOC associations differ according to one's general expectations regarding the impact of eating on emotions. Fischer et al. (82) found that women with BN or subthreshold BN who more strongly believed that eating would relieve NA had a *smaller* increase in NA in the hours prior to OBEs, and a smaller decrease in NA in the hours after OBEs. Though somewhat counterintuitive, findings are consistent with expectancy theory in suggesting that individuals who more strongly expect eating to relieve negative emotions may require less of an affective “push” to engage in LOC.

Intriguingly, studies examining *within-person* changes in eating expectancies show a different pattern of effects. In the two studies that examined within-person changes in eating expectancies among individuals with recurrent OBEs (48, 53), OBEs were most likely to occur following moments when both NA and the belief that eating would relieve negative emotions were higher than average for a given individual. In other words, LOC was most likely when a person was feeling intense negative emotions at a given moment in time, and believed that eating would help them feel better in that situation. A three-way interaction between eating expectancies, NA, and dietary restraint in Pearson et al. (53) suggested that the likelihood of OBEs when experiencing NA was amplified even more if a person was also trying to restrict their food intake.

When between- and within-person effects of eating expectancies are considered together, they suggest that people who generally believe that eating will help them feel better will engage in LOC more readily when experiencing even a small increase in negative emotions. Simultaneously, all people may be at increased risk of LOC during moments when NA, dietary restraint, and beliefs that eating would help them feel better are particularly intense. Notably, this pattern of effects is consistent with the predictions of expectancy theory, as well as some elements of restraint theory.

#### Negative Urgency/Impulsivity

Four studies examined whether facets of trait impulsivity were associated with different trajectories of NA surrounding LOC in women with AN (89), women with BN (44, 82), or women with regular OBEs [who may not have met criteria for any ED diagnosis (50)]. Three of these studies found that self-reported overall impulsivity (44) or impulsivity in response to negative emotions specifically [i.e., negative urgency (82, 89)] were associated with smaller increases in negative emotions prior to LOC. Culbert et al. (89) and Fischer et al. (82) also found smaller decreases in NA following LOC for women who were high in negative urgency. Results suggest that smaller increases in NA may be required to trigger LOC in individuals who tend to act impulsively in response to negative feelings.

In contrast, Smith et al. (61) found that NA was *more strongly* associated with self-defined binge eating at the next signal among women who showed greater delay discounting on a Monetary Choice Questionnaire (indicating a preference for smaller, more immediate rewards over larger, delayed rewards). In making sense of these findings, it is important to note that behavioral and questionnaire measures of impulsivity are only modestly correlated [*r*s ~ −0.10 to 0.25 (110)], suggesting that these methods measure relatively distinct constructs. It may ultimately be that smaller increases in NA trigger LOC for people who *believe* they cannot tolerate NA without acting, regardless of their actual capacity for inhibitory control.

## Discussion

Experience sampling research to date presents evidence of complex associations between NA and LOC in daily life that do not completely adhere to the predictions of the standard affect regulation model of LOC. Across study designs, elevated or increasing NA is associated with increased risk for LOC on a given day (30, 31, 33) and in subsequent hours [e.g., (43–45, 48, 50, 52–54, 56), perhaps particularly for individuals with frequent and recurrent LOC (109) and OBEs (37, 79, 92). However, the preponderance of evidence suggests that LOC may not be an effective strategy for decreasing NA, either immediately after the behavior or across longer timespans. Designs that have measured NA immediately before and after LOC eating have consistently found that NA is greater after LOC than before LOC (76–79, 94). While NA begins to decrease in the hours following LOC (e.g., (64, 80–85, 87)], research has often found that it remains elevated over baseline (64, 65, 90), and that LOC on 1 day tends to predict higher NA on the next (30, 31). Purging after LOC may decrease NA, but only to levels observed before LOC (102, 104). Overall, results from experience sampling studies challenge the view that LOC is maintained primarily through its ability to reduce negative emotions.

Even if LOC does not improve mood, people who generally *expect* to feel better after eating may be particularly vulnerable to LOC when they experience even small increases in NA (82). Greater expectations that eating will help one feel better in a given moment also strengthen the association between NA and LOC a short time later (48, 53). Expectations that eating will help one feel better may be particularly likely to lead to LOC when combined with other state risk factors for LOC, such as food restriction (53). Research findings to date are therefore consistent with expectancy theory, and highlight the potential role of cognitive factors (e.g., beliefs about the affective consequences of eating) in the maintenance of LOC. Interestingly, the two-way interaction between dietary restraint and NA was not significant in Pearson et al. (53), suggesting that restraint may only amplify the association between NA and subsequent LOC when a person also believes that eating will help them feel better. However, additional studies are needed to more fully examine whether restraint might weaken cognitive control in the presence of NA and contribute to LOC through the mechanisms proposed by restraint theory.

If people often feel worse after LOC, why does this behavior persist? Escape theory and trade-off theory offer two potential explanations by proposing that NA and self-critical thoughts may decrease *during* LOC (23), or that LOC may replace one negative emotion with another that is less aversive (25). Findings from experience sampling research have been mixed for both these theories. While some studies have found that NA temporarily decreases during LOC (100, 101), an equal number have found that NA remains constant or increases across LOC (102, 103). One study suggests that people with greater overall dissociative tendencies experience more rapid decreases in NA after LOC (87). However, Mason et al. (87) did not examine emotions or dissociation during LOC itself, and it therefore remains unclear whether a dissociative experience during LOC may temporarily reduce NA, as predicted by escape theory. Rather than replacing more aversive negative emotions with less unpleasant ones, LOC seems most likely to decrease low arousal negative emotions (e.g., boredom) and increase high arousal emotions that may be more distressing (e.g., guilt, anger at self) (101, 104). Neither theory therefore provides an unambiguous explanation for the persistence of LOC, at least based on current research.

One possible alternative explanation is that people with LOC may mistakenly attribute the gradual decrease in NA following LOC to the act of eating itself. People with EDs are more likely than people without disordered eating to endorse maladaptive beliefs about emotions, such as the idea that emotions will last forever or become overwhelming (111). In other words, individuals with LOC may fear (at least in the moment, when negative emotions are intense) that they will *never* feel better if they do not engage in LOC, or at least do something to regulate their affect. Interestingly, similar maladaptive beliefs about emotions are common in anxiety disorders, such as the belief that emotions will become so strong that they cannot be tolerated in panic disorder (112). Individuals with these beliefs may fail to recognize that negative emotions will eventually decrease on their own, and could potentially misattribute the natural decrease in NA over time following a distressing event to LOC. To test this theory, it would first be necessary to definitively establish that the decrease in NA following LOC is in fact no faster than the decrease in NA we would expect in the hours after a peak in negative emotions (e.g., after an unpleasant event) not accompanied by LOC. It would also be informative to conduct additional research on the beliefs about emotions endorsed by people directly before and after LOC (e.g., the extent to which individuals believe they will feel better on their own over time if they do nothing, the extent to which they believe LOC helped them feel better). If the evidence suggests that LOC does not hasten a decline in NA, but people with LOC strongly believe that they must act to feel better, individuals with LOC may benefit from psychoeducation and experiential exercises demonstrating that NA will frequently decrease over time on its own, even if they do nothing at all.

A complementary potential explanation is that, unless explicitly asked, people with LOC may not pay close attention to how their emotions change pre- to post-eating. Alexithymia, or difficulty identifying and communicating feelings, is common among people with OBEs (113) and other forms of LOC (114). If a person is not closely attuned to how their emotions change when they experience LOC, it may be easier to maintain the belief that LOC decreases NA, even when this is not the case. This may be particularly true in a cultural context where social messages about the soothing effects of eating (e.g., “comfort food”) are ubiquitous (115), and the “default belief” may be that eating helps reduce NA. In this case, mindfulness of one's own emotional experience, and mindful attention to how that experience may differ from common cultural beliefs, could potentially help decrease a person's tendency to engage in LOC to relieve NA.

It is also possible that the effects of LOC on NA may change over time. Specifically, LOC may effectively decrease NA early in the course of an illness such as BN or BED, but begin to increase NA as LOC becomes more frequent or persistent, and its associated undesirable consequences become clearer. LOC may then persist as a habitual response to NA that becomes difficult to change even though it is no longer reinforcing. Additional research on individuals who have recently begun experiencing LOC (perhaps recruited from high-risk populations or large, population-based samples of adolescents or young adults) vs. those who have experienced this behavior for months or years could illuminate differences in the emotional impact of LOC across time.

Affect regulation theories of LOC have traditionally focused on changes in NA, with less attention to the effects of LOC on positive affect (PA; e.g., joy, excitement, or contentment). Note that PA is not merely the absence of negative emotions—correlations between well-validated measures of NA and PA are small [e.g., −0.12 to −0.23 on the Positive and Negative Affect Schedule (105)]. It is therefore possible that someone could feel worse (i.e., more NA) *and better* (i.e., more PA) after LOC. This is particularly true given that consumption of palatable foods is intrinsically pleasurable, especially after a period of food restriction (116). Most studies that have examined NA and PA separately have found that PA is lower or unchanged immediately after LOC (65, 70, 74), arguing against maintenance of LOC via positive reinforcement [but see (104), who found an increase in happiness/relief following purging]. However, relatively few studies have investigated PA, and additional research on changes in PA surrounding LOC in daily life is warranted. In particular, it is possible that increases in PA following LOC may be particularly likely if LOC was proceeded by dietary restriction. This possibility could be investigated by examining prior restriction as a moderator of increases in PA following LOC in future research.

Three additional limitations of existing experience sampling research on associations between NA and LOC should be acknowledged. A first key limitation is inherent to the methodology—namely, that intensive monitoring of NA surrounding LOC may itself alter NA-LOC associations. If one function of LOC is to escape awareness of NA, drawing attention to emotions may negate this effect. This is particularly true for more intensive EMA designs that sample affect at multiple timepoints during LOC itself. One potential solution is to incorporate affect measures into future EMA research that do not require active reporting by participants. While physiological sensations do not correlate perfectly with verbal reports of emotion (117), non-invasive monitoring of heart rate or skin conductance across the day may provide some insight into changes in emotional arousal before and after LOC without explicitly drawing a person's attention to their emotional state. Emerging research also suggests that it may be possible to detect some changes in mood through passive monitoring of other aspects of a person's behavior, such as the speed or pitch of their speech [which can be recorded throughout the day using cellphones (118)].

A second limitation relates to who has been included in experience sampling research on LOC. The vast majority of studies, particularly studies with clinical populations, have only included women. Among studies that have included men, none have examined whether associations between NA and LOC differ across gender. Relatedly, most studies have used participant samples that are predominantly (often over 95%) white. This is despite the fact that LOC is equally or more common among people of color than among white individuals (119). Additional research is therefore urgently needed to examine whether associations between NA and LOC are similar across gender and race/ethnicity. Differences across ethnicity are particularly important to investigate further because some factors that moderate NA-LOC eating associations, such as eating expectancies, may be based in sociocultural norms that could vary across diverse groups.

A third limitation is that experience sampling research on NA-LOC relationships has thus far paid little attention to biological and environmental contextual factors that might impact NA, LOC, or their association at a given moment in time. Emotions, and adaptive responses to emotions, are intimately tied to the context in which they occur (120). Both environmental and biological factors can influence the intensity and persistence of negative emotions, and may also impact the extent to which they lead to LOC. To date, the vast majority of experience sampling research on LOC has paid little attention to the impact of a person's momentary environment (e.g., whether someone is alone or with others, the presence of palatable food or messages stigmatizing larger bodies) on nuances of emotions or behavior. Relatedly, almost no research has examined the biological context in which negative emotions and LOC occur. Biological factors that may be outside of a person's immediate awareness (e.g., menstrual cycle phase, blood sugar levels, fatigue) may increase NA, or reactivity to negative events, in a way that increases risk for LOC. A more detailed understanding of the moment-to-moment environmental and biological factors that may increase NA or strengthen NA-LOC associations is needed to refine and clarify affect regulation models. For example, understanding the impact of biological and environmental moderators on NA-LOC associations could inform our understanding of why LOC might persist even if it is not immediately reinforcing on average (i.e., whether it is intermittently reinforcing based on the context). Experience sampling research is uniquely poised to collect information about momentary biological and environmental factors before, during, and after LOC in ecologically valid settings. When combined with other minimally invasive measures (e.g., saliva collection for hormone levels, heart rate monitoring), it has the potential to help us identify with precision when and why NA may trigger maladaptive eating behavior. In the future, additional research in other cultural contexts could also help illuminate the influence of broader cultural factors and beliefs on NA-LOC relationships, and ensure that research to date generalizes to diverse populations.

**Figure 2:**
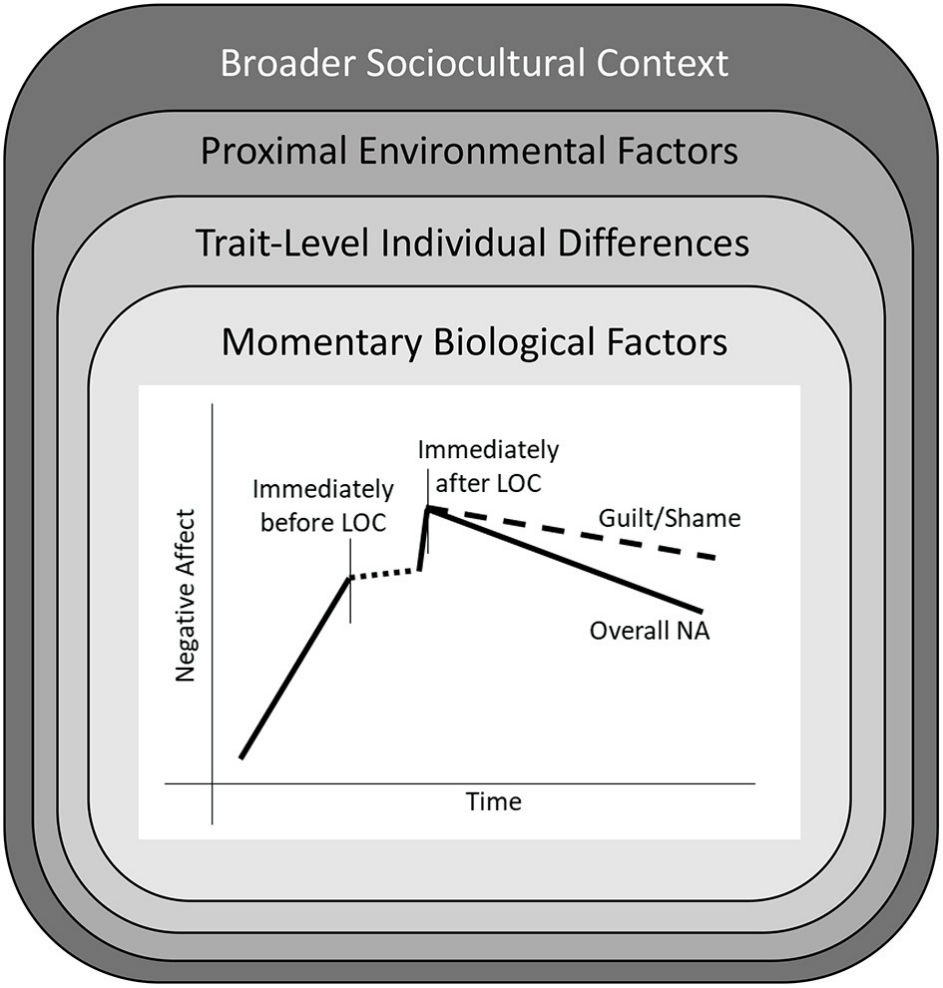
A revised affect-focused model of loss of control eating (LOC). The central figure shows the nomothetic pattern of changes in negative affect (NA) surrounding LOC found in a majority of experience sampling studies. This model is nestled within several layers of biological and environmental factors that could potentially alter the strength of NA-LOC associations. First are momentary biological factors, such as changes in hormone levels and blood sugar, that may alter reactivity to unpleasant events or stimuli. Second are trait-level individual differences that may moderate NA-LOC associations. Third are proximal environmental factors, such as interactions with peers or family members, that may increase (or decrease) NA, or affect the likelihood that NA will lead to LOC. Finally, individuals are encompassed by a broader sociocultural context.

A proposed, revised affect-focused model of LOC is depicted in Figure 2. Figure 2 reflects existing research showing that LOC is proceeded by an increase in NA, and followed by an immediate increase and gradual decline in NA. The dashed line during the period of LOC represents uncertainty regarding what happens to emotions while LOC is in progress, and the need for additional creative research to examine this phase (e.g., using passive monitoring of heart rate or vocal tone). While overall NA is shown to decrease in the hours following LOC, a separate line is depicted for guilt/shame, which evidence suggests remains elevated even after other negative emotions begin to subside. This nomothetic pattern of NA surrounding LOC is qualified by several layers of biological and environmental factors that may either directly increase NA, or alter the strength of NA-LOC associations. These factors include a person's momentary biological state, trait-level individual differences (e.g., negative urgency, beliefs about the effects of eating on emotions), momentary proximal environmental factors, and the broader cultural context. Which of these factors most strongly influence the pattern of effects shown in the central graph in Figure 2, or how they might interact with each other, represents an exciting area for future research.

Of note, the proposed affect-focused model in Figure 2 shares some similarities with conceptualizations of other forms of psychopathology. Most notably, it is similar to models of non-suicidal self-injury (NSSI), in which negative emotions may drive a person to injure themselves with (in many cases) the ultimate goal of feeling better. As with LOC, people frequently report engaging in NSSI to relieve NA (121). However, evidence from experience sampling research is mixed with regards to whether NA actually decreases following NSSI in daily life (122), with some research finding that NA peaks shortly after NSSI and slowly decreases over subsequent hours (123). As with LOC, motivations for engaging in NSSI may be complex, with elements of wanting to feel better (e.g., release difficult emotions) and wanting to feel worse (e.g., punish self for not meeting one's standards) (121). Engaging in NSSI may also increase PA, similar to consumption of palatable food in LOC (124). These similarities suggest that similar underlying mechanisms may be at play with respect to NSSI and LOC, whereby difficulty tolerating and managing distress leads to maladaptive behavior in a desire to feel better (even if this behavior is not always effective, and there are negative longer term consequences).

Conceptual similarities between the proposed affect-focused model of LOC and NSSI suggest that similar treatment approaches may be appropriate. In particular, DBT has shown promise for both NSSI and disorders characterized by LOC by targeting underlying difficulties with distress tolerance (i.e., the ability to experience NA without engaging in maladaptive behavior), problem solving, and emotion regulation (125). However, while LOC may function similarly to NSSI with respect to emotion regulation, it may also differ in some important respects. In particular, LOC may be propelled not only by NA, but also by biological drives to consume food after food restriction. LOC may therefore require additional treatment elements beyond simply improving distress tolerance and emotion regulation, such as implementing regular, balanced meals, as occurs in CBT for EDs (4). Because consumption of palatable foods during LOC may be pleasurable, it may also be necessary to incorporate therapeutic elements to increase experience of PA, rather than just decreasing or improving tolerance for NA.

While additional work remains to be done, research to date nevertheless offers some practical clinical implications. Evidence that elevated NA increases risk for subsequent LOC supports key aspects of existing CBT-based treatments, such as working with clients to identify the situations that are most likely to trigger NA and develop strategies to help regulate their emotions at these times (4). Adding DBT-based techniques from protocols adapted for BED and BN (20) that teach distress tolerance (e.g., distraction, self-soothing) and problem solving (e.g., weighing pros and cons) skills may have added benefit in helping individuals develop the tools necessary to abstain from LOC when feeling negative emotions. Clients may also benefit from behavioral experiments that challenge the belief that LOC decreases NA. For example, clients could be asked to record how they feel immediately before and after LOC, and these data could be used to challenge ideas about the emotion regulatory properties of LOC. Finally, mindfulness-based exercises and emotional exposures [i.e., exercises to build acceptance and tolerance of emotions, as described in Barlow's Unified Protocol (126)] could be used to help decrease reactivity to NA and help clients learn that negative emotions decrease on their own, even without LOC or other maladaptive behaviors intended to numb these feelings. Exposure and response prevention (ERP) for obsessive-compulsive disorder (127) offers some complementary techniques. In traditional ERP for OCD, clients are coached to refrain from engaging in compulsions when experiencing obsessive thoughts so that they may learn that compulsions are not necessary for reducing anxiety and avoiding catastrophic outcomes. Analogously, clients with LOC could be intentionally exposed to situations (real or imaginal) that provoke NA, and coached to refrain from engaging in maladaptive eating behavior to learn that this behavior is not necessary for feeling better. Similar techniques have already been effectively incorporated to some extent in existing cue exposure and response prevention treatments for LOC (128), though the focus has more often been on exposure to environmental factors associated with LOC (e.g., seeing a particular food, being in a particular room in the house) than affective factors that may trigger this behavior.

Though the standard affect regulation model may require some revision, experience sampling research has affirmed the importance of NA as a driver of LOC in daily life. Future research that incorporates individual differences and momentary biological and environmental factors will help us continue to expand our understanding of when, why, and for whom negative feelings may trigger dysregulated eating.

## Author Contributions

The author confirms being the sole contributor of this work and has approved it for publication.

## Funding

This work was supported by a grant from the National Institute of Mental Health (R01 MH111715-03S1). The content is solely the responsibility of the author and does not necessarily represent the official views of the National Institute of Mental Health.

## Conflict of Interest

The author declares that the research was conducted in the absence of any commercial or financial relationships that could be construed as a potential conflict of interest.

## Publisher's Note

All claims expressed in this article are solely those of the authors and do not necessarily represent those of their affiliated organizations, or those of the publisher, the editors and the reviewers. Any product that may be evaluated in this article, or claim that may be made by its manufacturer, is not guaranteed or endorsed by the publisher.
