# Supplementary material for: Affect Dysregulation in Context: Implications and Future Directions of Experience Sampling Research on Affect Regulation Models of Loss of Control Eating
Source: Front Psychiatry. 2021 Sep 27;12:747854. doi: 10.3389/fpsyt.2021.747854 (PMC8502879; doi:10.3389/fpsyt.2021.747854)
Supplement: Supplementary file 1 [file Table_1.DOCX]

**Appendix A.** Demographic information, methodology, and key findings from included studies

| ***Daily Dairy Studies*** | | | | | |
| --- | --- | --- | --- | --- | --- |
| **Citation** | **Sample Size and Study Demographics** | **Measures of Negative Affect (NA) and LOC Eating** | **Sampling Frequency** | **Key Findings** | **Overall Study Quality** |
| Barker, E. T., Williams, R. L., & Galambos, N. L. (2006). Daily spillover to and from binge eating in first-year university females. *Eating Disorders*, *14*(3), 229-242. | 66 first-year undergraduate women at a large Canadian university recruited through an introductory psychology research pool. Mean age = 19 years (*SD* = 1.11 years), race/ethnicity not reported. | NA measured using the PANAS. LOC eating measured using 7 true/false items assessing binge eating behaviors or symptoms based on the EDI. | Once per day for two weeks. | At a between-person level, greater average levels of NA were associated with greater average binge eating symptoms over the 14 days. Participants were more likely to experience symptoms of binge eating on days characterized by higher NA. Binge eating symptoms on day 1 predicted higher NA on day 2, but day 1 NA did not predict day 2 binge eating. | Low, due to convenience sample, low incidence of binge eating events, some shortcomings in reporting of results (e.g., no CI). |
| Haedt‐Matt, A. A., Keel, P. K., Racine, S. E., Burt, S. A., Hu, J. Y., Boker, S.,...Klump, K. L. (2014). Do emotional eating urges regulate affect? Concurrent and prospective associations and implications for risk models of binge eating. *International Journal of Eating Disorders*, *47*(8), 874-877. | 239 female twins ages 16-25 (mean = 17.79) from the Michigan State University Twin Registry. 83.2% white; 12.6% Black/African American; 4.2% Latina. Mean BMI = 23.98. | NA measured using the PANAS. Emotional eating measured using the Dutch Eating Behavior Questionnaire Emotional Eating scale. | Once per day for 45 consecutive days. | Greater NA and lower PA were associated with greater levels of emotional eating on the same day. However, neither day 1 NA nor PA predicted day 2 emotional eating. Conversely, greater day 1 emotional eating predicted greater day 2 NA (but not PA). | High, due to representative, population-based sample, well-powered, extended sampling period, well-validated measures. |
| Haynos, A. F., Crosby, R. D., Engel, S. G., Lavender, J. M., Wonderlich, S. A., Mitchell, J. E.,...Le Grange, D. (2015). Initial test of an emotional avoidance model of restriction in anorexia nervosa using ecological momentary assessment. *Journal of Psychiatric Research*, *68*, 134-139. | 118 women with AN (see Berg et al., 2017). | NA measured using a modified version of the PANAS (nervous, disgusted, distressed, ashamed, angry at self, afraid, sad, and dissatisfied with self). Tension and anxiety were also measured using the POMS. Binge eating defined as “an amount of food that you consider excessive, or an amount of food that other people would consider excessive, with an associated loss of control or the feeling of being compelled to eat”. | Six times per day for two weeks, plus event contingent ratings made directly after eating. Analyses averaged across the day to examine daily patterns. | Days with binge eating were characterized by higher NA and anxiety/tension than days characterized by restriction alone or neither restriction nor binge eating. | High, due to adequate power, an appropriate (though non-fully representative) sample, good sampling frequency/duration, adequately validated measures, adequate reporting of results. |
| Mason, T. B., Heron, K. E., Braitman, A. L., & Lewis, R. J. (2016). A daily diary study of perceived social isolation, dietary restraint, and negative affect in binge eating. *Appetite*, *97*, 94-100. | 54 undergraduate women who reported engaging in at least one binge eating episode in the past week (ages 18-49 years, mean = 24.98). Mean BMI = 26.65. 57.3% white, 25.9% Black/African American 11.1% multiracial, 1.9% Asian, 1.9% Native Hawaiian or Pacific Islander, 1.9% other race/ethnicity. | NA measured using the short version of the PANAS (distressed, upset, ashamed, nervous, afraid). Binge eating measured using items from the Eating Disorder Inventory Bulimia scale and the Eating Disorder Diagnostic Scale. | Once per day for two weeks. | Participants reported more binge eating symptoms on days characterized by higher NA, controlling for dietary restraint. The interaction between within-person NA and within-person dietary restraint was not significant. After controlling for social isolation, the association between NA and binge eating symptoms was no longer significant. | Low, due to convenience sample, high dropout rates. |
| Mikhail, M. E., & Kring, A. M. (2019). Emotion regulation strategy use and eating disorder symptoms in daily life. *Eating Behaviors*, *34*, 101315. | 53 undergraduate women (ages 18-41, mean = 20.90). 62.3% Asian, 30.2% white, 1.9% multiethnic, 5.7% other ethnicity, 11.3% Latina. Mean BMI = 22.26 | NA measured using items derived from the circumplex model of emotions (sad, guilty, irritable, nervous, scared, bored, angry, embarrassed, lonely). Binge eating measured using a single item assessing overeating and LOC. | NA measured four times per day and binge eating measured once per day for one week. | Daily NA was not significantly associated with odds of binge eating on a given day, but the direction of the effect was positive (i.e., greater odds of binge eating on days with higher NA). | Low, due to convenience sample, limited sampling duration, somewhat limited validation of study measures. |
| Sherry, S. B., Sabourin, B. C., Hall, P. A., Hewitt, P. L., Flett, G. L., & Gralnick, T. M. (2014). The perfectionism model of binge eating: Testing unique contributions, mediating mechanisms, and cross-cultural similarities using a daily diary methodology. *Psychology of Addictive Behaviors*, *28*(4), 1230-1239. | 566 undergraduate women. Mean age = 19.53. Mean BMI = 21.06. 90.8% Asian Canadian or European Canadian. | Depressive affect measured using items from the POMS and Depression Adjective Check Lists. Anxious affect measured using items from the POMS, Cognitive Worry Scale, and Autonomic Emotional Subscale. Binge eating assessed using items from the Bulimia Test-Revised, Eating Disorder Inventory Bulimia subscale, and the binge eating subscale of the Eating Disorder Diagnostic Scale. | Once per day for one week. | Daily depressive (but not anxious) affect was associated with daily binge eating symptoms after controlling for dietary restraint and interpersonal factors. No significant differences in this association were observed between Asian Canadian and European Canadian participants. | Medium, due to use of a convenience sample and somewhat brief sampling duration, but adequate power, validation of study measures, and reporting of results. |
| Turner, B. J., Yiu, A., Claes, L., Muehlenkamp, J. J., & Chapman, A. L. (2016). Occurrence and co-occurrence of nonsuicidal self-injury and disordered eating in a daily diary study: Which behavior, when? *Psychiatry Research*, *246*, 39-47. | 60 adults who reported repeated episodes of non-suicidal self-injury (NSSI) (92% women). Ages 18-35 (mean age = 23.13). 68% white, 12% Asian, 12% South Asian, 8% other races/ethnicities. | Mood measured using the Multidimensional Mood Questionnaire. Participants also reported whether they had felt scared/anxious, numb/nothing, sad/worthless, angry at self, self-hatred, angry at others, rejected/hurt, or overwhelmed before engaging in behaviors. Binge eating measured using a single item assessing overeating and LOC. | Once per day for 14 days. Participants were asked to retrospectively report on their mood during the morning, afternoon, and evening at the end of the day. | Participants reported being more likely to act on binge eating or purging thoughts when those thoughts were proceeded by feelings of self-hatred. Overall mood on days characterized by NSSI and binge eating/purging was similar, except that participants reported feeling more intense fatigue in the morning on days characterized by NSSI. | High, due to generally adequate power, adequately validated study measures, adequate reporting, and a diverse and appropriate (though not fully representative) sample. |
| ***Ecological Momentary Assessment Studies of the Hours Surrounding LOC Eating*** | | | | | |
| **Citation** | **Sample Size and Study Demographics** | **Measures of NA and LOC Eating** | **Sampling Frequency** | **Key Findings** | **Overall Study Quality** |
| Alpers, G. W., & Tuschen-Caffier, B. (2001). Negative feelings and the desire to eat in bulimia nervosa. *Eating Behaviors*, *2*(4), 339-352. | Women with BN (*n* = 40), panic disorder (*n* = 40), or no psychiatric disorder (*n* = 40). Ages 17-54 (mean age = 24.8 for BN group, 34.0 for panic disorder group, 24.9 for control group). Mean BMI = 20.4 for BN group, 22.2 for panic disorder group, 20.6 for control group. Race/ethnicity not reported. | Individual negative emotions were rated on a Likert scale (tension, sadness, anxiety, insecurity) throughout the day. Women with BN rated their global mood from 1 (positive mood) to 7 (bad mood) before binge eating, after binge eating, and after purging. LOC eating not clearly defined. | Every waking hour for 2 consecutive days. | Participants with BN reported greater tension and insecurity in the hour before binge eating than during the rest of the day. Levels of anxiety and sadness were also higher in the hour before binge eating, but these effects were not statistically significant. Global mood was significantly worse after binge eating than before binge eating, but returned to pre-binge eating levels after purging. | Low, due to short sampling duration, lack of validation of the global mood rating, an unclearly defined measure of LOC eating. |
| Ambwani, S., Roche, M. J., Minnick, A. M., & Pincus, A. L. (2015). Negative affect, interpersonal perception, and binge eating behavior: An experience sampling study. *International Journal of Eating Disorders*, *48*(6), 715-726. | 40 undergraduate women with at least one objective binge eating episode in the past month and a global EDE-Q score at least 1 SD above community norms. Ages 18-23 (mean age = 19.22). Mean BMI = 26.65. 85% white, 2.5% Black/African American, 5% Latina, 2.5% Native American, 5% multiracial. | NA measured using 10 items from the PANAS extended form with high factor loadings and theoretical relevance (afraid, lonely, irritable, ashamed, disgusted, nervous, dissatisfied with self, jittery, sad, angry with self). Participants given a definition of binge eating, and asked to indicate whether study eating episodes involved overeating and LOC. | Six times per day for two weeks. | Greater NA was associated with greater odds of binge eating at the next signal. The association between NA and binge eating was greater for individuals who reported more cold and dominant interpersonal problems at baseline. | Medium, due to low power, but adequate study measures, sampling duration/frequency, and reporting of results. |
| Becker, K. R., Fischer, S., Crosby, R. D., Engel, S. G., & Wonderlich, S. A. (2018). Dimensional analysis of emotion trajectories before and after disordered eating behaviors in a sample of women with bulimia nervosa. *Psychiatry Research*, *268*, 490-500. | 133 women with BN. Ages 18-55 (mean age = 25.3). Mean BMI = 23.9. 85.5% with lifetime mood disorder, 58.6% with lifetime anxiety disorder. 95.5% white. | NA measured using a modified version of the PANAS and items from the POMS, divided into high arousal, avoidant NA; high arousal, approach NA; low arousal, avoidant NA. Binge eating defined as “an amount of food that you consider excessive, or an amount of food that other people would consider excessive, with an associated loss of control or the feeling of being compelled to eat”. | Six times per day for two weeks. | All negative emotion dimensions increased linearly prior to binge eating and decreased linearly following binge eating. High arousal, avoidant NA was greater than high arousal, approach NA both before and after binge eating. | High, due to adequate power, an appropriate (though non-fully representative) sample, good sampling frequency/duration, adequately clear and validated measures, adequate reporting of results. |
| Berg, K. C., Cao, L., Crosby, R. D., Engel, S. G., Peterson, C. B., Crow, S. J.,...Wonderlich, S. A. (2017). Negative affect and binge eating: Reconciling differences between two analytic approaches in ecological momentary assessment research. *International Journal of Eating Disorders*, *50*(10), 1222-1230. | 118 women with AN and 131 women with BN (BN sample overlaps with Becker et al., 2018). Mean age = 25.3 in both AN and BN groups. Mean BMI for AN group = 17.2, for BN group = 23.2. AN group 96.0% white, BN group 96.9% white. | NA measured using modified versions of the PANAS (for AN group, nervous, disgusted, distressed, ashamed, angry at self, afraid, sad, and dissatisfied with self; for BN group, afraid, lonely, irritable, ashamed, disgusted, nervous, dissatisfied with self, jittery, sad, angry at self, and distressed). Binge eating defined as “an amount of food that you consider excessive, or an amount of food that other people would consider excessive, with an associated loss of control or the feeling of being compelled to eat”. | Six times per day for two weeks, plus event contingent ratings made directly after eating. | The most proximal post-binge eating rating of NA was greater than the most proximal pre-binge eating rating of NA (indicating higher NA after binge eating than before binge eating). However, the most proximal post-binge eating rating was made closer in time to the binge (~20 minutes after) than the most proximal pre-binge eating rating (~2.5 hours before). The trajectory of NA was increasing before binge eating, and decreasing following binge eating. | High, due to adequate power, an appropriate (though non-fully representative) sample, good sampling frequency/duration, adequately validated measures, adequate reporting of results. |
| Berg, K. C., Crosby, R. D., Cao, L., Crow, S. J., Engel, S. G., Wonderlich, S. A., & Peterson, C. B. (2015). Negative affect prior to and following overeating‐only, loss of control eating‐only, and binge eating episodes in obese adults. *International Journal of Eating Disorders*, *48*(6), 641-653. | 50 obese adults (84% women). Ages 21-64 (mean = 43.0). Mean BMI = 40.3. 76% white, 14% Black/African American, 6% Asian, 4% other race/ethnicity. Participants could not have lifetime AN or BN. | NA measured with a modified version of the PANAS (afraid, nervous, jittery, ashamed, disgusted, dissatisfied with self, angry with self, irritable, angry, lonely, sad). Participants rated the extent to which they felt they had overeaten (1 item) and felt LOC (4 items) for each eating episode. Binge eating defined as a score of ≥ 3 on the overeating item and at least one LOC item. | Six times per day for two weeks, plus event contingent ratings made directly after eating. | The trajectory of global NA was increasing in the hours prior to binge eating, and decreasing in the hours following binge eating. When emotion facets (i.e., sadness, anxiety, anger, and guilt) were examined separately, only guilt-related emotions were on an increasing trajectory prior to binge eating, and a decreasing trajectory following binge eating. The trajectory of negative emotions did not significantly change surrounding LOC-only episodes not characterized by overeating. | High, due to well defined and validated measures, appropriate sampling frequency and duration, adequate reporting of results, and an appropriate community sample. Though power was somewhat low, this was adequately acknowledged in the paper. |
| Berg, K. C., Crosby, R. D., Cao, L., Peterson, C. B., Engel, S. G., Mitchell, J. E., & Wonderlich, S. A. (2013). Facets of negative affect prior to and following binge-only, purge-only, and binge/purge events in women with bulimia nervosa. *Journal of Abnormal Psychology*, *122*(1), 111-118. | 133 women with BN (see Becker et al., 2018). | NA measured using a modified version of the PANAS (afraid, lonely, irritable, ashamed, disgusted, nervous, dissatisfied with self, jittery, sad, angry at self, and distressed). Binge eating defined as “an amount of food that you consider excessive, or an amount of food that other people would consider excessive, with an associated loss of control or the feeling of being compelled to eat”. | Six times per day for two weeks, plus event contingent ratings made directly after eating. | All four facets of NA derived from the PANAS (fear, guilt, hostility, sadness) were on an increasing trajectory prior to binge eating, and a decreasing trajectory following binge eating. When all forms of NA were included in the same model, only changes in the trajectory of guilt were statistically significant. | High, due to adequate power, an appropriate (though non-fully representative) sample, good sampling frequency/duration, adequately clear and validated measures, adequate reporting of results. |
| Berg, K. C., Peterson, C. B., Crosby, R. D., Cao, L., Crow, S. J., Engel, S. G., & Wonderlich, S. A. (2014). Relationship between daily affect and overeating-only, loss of control eating-only, and binge eating episodes in obese adults. *Psychiatry Research*, *215*(1), 185-191. | 50 obese adults (84% women; see Berg et al., 2015). | NA measured with a modified version of the PANAS (afraid, nervous, jittery, ashamed, disgusted, dissatisfied with self, angry with self, irritable, angry, lonely, sad). Participants rated the extent to which they felt they had overeaten (1 item) and felt LOC (4 items) for each eating episode. Binge eating defined as a score of ≥ 3 on the overeating item and at least one LOC item. | Six times per day for two weeks, plus event contingent ratings made directly after eating. | Relative to days characterized by low stable NA, binge eating was more common on days characterized by stable moderate NA, stable high NA, late increasing NA, gradually increasing NA, and U-shaped NA. There were no differences in rates of LOC-only between days characterized by low stable NA and days with other NA patterns. | High, due to well defined and validated measures, appropriate sampling frequency and duration, adequate reporting of results, and an appropriate community sample. Though power was relatively low, this was adequately acknowledged in the paper. |
| Cooper, P. J., & Bowskill, R. (1986). Dysphoric mood and overeating. *British Journal of Clinical Psychology*, *25*(2), 155-156. | Individuals entering treatment for BN (sample size not provided). Mean age = 18.3. Race/ethnicity not provided. | NA measured using individual emotion items (depressed, anxious, lonely, bored) rated on visual analogue scales. LOC eating not clearly defined. | Ratings of mood made hourly for one week. | Ratings of depression, anxiety, loneliness, and boredom were significantly greater during the three hours proceeding bulimic episodes than other times of day. Anxiety and loneliness ratings remained significantly elevated after bulimic episodes. | Low, due to inadequate reporting of sample characteristics (including sample N) and an unclear definition of LOC eating. |
| Crosby, R. D., Wonderlich, S. A., Engel, S. G., Simonich, H., Smyth, J., & Mitchell, J. E. (2009). Daily mood patterns and bulimic behaviors in the natural environment. *Behaviour Research and Therapy*, *47*(3), 181-188. | 133 women with BN (see Becker et al., 2018). | NA measured using a modified version of the PANAS (afraid, lonely, irritable, ashamed, disgusted, nervous, dissatisfied with self, jittery, sad, angry at self, and distressed). Binge eating defined as “an amount of food that you consider excessive, or an amount of food that other people would consider excessive, with an associated loss of control or the feeling of being compelled to eat”. | Six times per day for two weeks. | Binge eating was most likely on days with early increasing NA, stable high NA, and U shaped NA. Binge eating was more likely later in the day. | High, due to adequate power, an appropriate (though non-fully representative) sample, good sampling frequency/duration, adequately clear and validated measures, adequate reporting of results. |
| Culbert, K. M., Lavender, J. M., Crosby, R. D., Wonderlich, S. A., Engel, S. G., Peterson, C. B.,...Fischer, S. (2016). Associations between negative affect and binge/purge behaviors in women with anorexia nervosa: Considering the role of negative urgency. *Comprehensive Psychiatry*, *66*, 104-112. | 82 women with AN. Ages 18-58 (mean age = 25.23). Mean BMI = 17.11. 97.6% white. | NA measured using a modified version of the PANAS (afraid, lonely, irritable, ashamed, disgusted, nervous, dissatisfied with self, jittery, sad, angry at self, and distressed). Binge eating defined as “an amount of food that you consider excessive, or an amount of food that other people would consider excessive, with an associated loss of control or the feeling of being compelled to eat”. | Six times per day for two weeks, plus event contingent ratings made directly after eating. | For women with low levels of negative urgency, binge eating was more frequent on days with high NA. Women with high negative urgency had equivalent levels of binge eating on days with high and low NA. Within day, the increasing trajectory of NA prior to binge eating, and the decreasing trajectory of NA following binge eating, were steeper for individuals with lower negative urgency. | High, due to adequate power, an appropriate (though non-fully representative) sample, good sampling frequency/duration, adequately clear and validated measures, adequate reporting of results. |
| Davis, R., Freeman, R. J., & Garner, D. M. (1988). A naturalistic investigation of eating behavior in bulimia nervosa. *Journal of Consulting and Clinical Psychology*, *56*(2), 273-279. | Treatment-seeking women with BN (*n* = 20), and women from the community without BN (*n* = 24). Mean age = 23.7 for BN group, 25.5 for non-BN group. Race/ethnicity not reported. | NA measured using a visual analogue scale from 0 (worst mood) to 100 (best mood). LOC eating not clearly defined. | Once per hour for six consecutive days. | Women with BN reported more negative mood in the hour prior to binge eating than in the hour prior to eating meals/snacks. | Low, due to low power, an unclearly defined measure of LOC eating, and inadequate validation of the affect measure. |
| Davis, R., Freeman, R., & Solyom, L. (1985). Mood and food: An analysis of bulimic episodes. In *Anorexia Nervosa and Bulimic Disorders* (pp. 331-335). Pergamon. | Treatment-seeking women with BN (*n* = 16), and women from the community without BN (*n* = 16). Mean age for women with BN = 23.6 (age not reported for control group). Race/ethnicity not reported. | NA measured using a visual analogue scale from 0 (worst mood) to 100 (best mood). LOC eating not clearly defined. | Once per hour for six consecutive days. | Participants with BN experienced worse than typical moods in the hour prior to bulimic episodes, which worsened further in the hour following bulimic episodes. A similar pattern was not observed for meals and snacks. | Low, due to low power, an unclearly defined measure of LOC eating, and inadequate validation of the affect measure. |
| De Young, K. P., Lavender, J. M., Wonderlich, S. A., Crosby, R. D., Engel, S. G., Mitchell, J. E.,...Le Grange, D. (2013). Moderators of post-binge eating negative emotion in eating disorders. *Journal of Psychiatric Research*, *47*(3), 323-328. | Women with BN (*n* = 121, mean age = 25.21) or AN (*n* = 47, mean age = 25.68). >95% white. | NA measured using 8 items from the PANAS-X (afraid, ashamed, disgusted, distressed, nervous, dissatisfied with self, sad, angry at self) chosen due to high factor loadings and relevance to EDs. Binge eating defined as “an amount of food that you consider excessive, or an amount of food that other people would consider excessive, with an associated loss of control or the feeling of being compelled to eat”. | Six times per day for two weeks, plus event contingent ratings made directly after eating. | Overall NA decreased in the first hour after binge eating. Reductions in overall NA following binge eating were similar for women with AN and BN. The shame-related facet of the PANAS also decreased in the hour following binge eating. Participants with BN experienced a greater decrease in guilt/shame following binge eating than participants with AN, and individuals who tended *not* to engage in self-induced vomiting experienced a greater decrease than those who did. | High, due to adequate power, an appropriate (though non-fully representative) sample, good sampling frequency/duration, adequately clear and validated measures, adequate reporting of results. |
| Elmore, D. K., & de Castro, J. M. (1990). Self‐rated moods and hunger in relation to spontaneous eating behavior in bulimics, recovered bulimics, and normals. *International Journal of Eating Disorders*, *9*(2), 179-190. | Women with current BN (*n* = 19), past BN (*n* = 12), and no ED (*n* = 21). Ages 18-36 (mean age = 22 for current BN group, 26 for past BN group, 26 for control group). Race/ethnicity not reported. | NA rated using bipolar elated-depressed and calm-anxious Likert scales. LOC eating not clearly defined. | Every time participants ate or drank for one week. | Depression increased across both meals and binge eating episodes for women with current BN. Pre-eating depression ratings did not significantly differ between meals and binge eating episodes for women with BN, but post-eating depression ratings were greater for binge eating episodes than for meals. Depression increased further after purging. | Low, due to low power, an unclearly defined measure of LOC eating, relatively high dropout rates (7/26 participants with BN dropped out). |
| Engel, S. G., Boseck, J. J., Crosby, R. D., Wonderlich, S. A., Mitchell, J. E., Smyth, J.,...Steiger, H. (2007). The relationship of momentary anger and impulsivity to bulimic behavior. *Behaviour Research and Therapy*, *45*(3), 437-447. | 133 women with BN (see Becker et al., 2018). | NA measured using the four-item anger-hostility subscale of the POMS. Binge eating defined as “an amount of food that you consider excessive, or an amount of food that other people would consider excessive, with an associated loss of control or the feeling of being compelled to eat”. | Six times per day for two weeks, plus event contingent ratings made directly after eating. | Greater anger averaged across observations prior to binge eating was associated with greater odds of subsequent binge eating. Anger intensity was less strongly associated with binge eating in participants with greater impulsivity at a trend level. | High, due to adequate power, an appropriate (though non-fully representative) sample, good sampling frequency/duration, adequately clear and validated measures, adequate reporting of results. |
| Engel, S. G., Wonderlich, S. A., Crosby, R. D., Mitchell, J. E., Crow, S., Peterson, C. B.,...Gordon, K. H. (2013). The role of affect in the maintenance of anorexia nervosa: Evidence from a naturalistic assessment of momentary behaviors and emotion. *Journal of Abnormal Psychology*, *122*(3), 709-719. | 118 women with AN (see Berg et al., 2017). | NA measured using a modified version of the PANAS (nervous, disgusted, distressed, ashamed, angry at self, afraid, sad, and dissatisfied with self). Binge eating defined as “an amount of food that you consider excessive, or an amount of food that other people would consider excessive, with an associated loss of control or the feeling of being compelled to eat”. | Six times per day for two weeks, plus event contingent ratings made directly after eating. | NA was greater at the first report after LOC eating than the last report before LOC eating. However, when looking at the trajectory of NA, NA was seen to rise prior to LOC eating and fall after LOC eating. | High, due to adequate power, an appropriate (though non-fully representative) sample, good sampling frequency/duration, adequately validated measures, adequate reporting of results. |
| Engelberg, M. J., Steiger, H., Gauvin, L., & Wonderlich, S. A. (2007). Binge antecedents in bulimic syndromes: An examination of dissociation and negative affect. *International Journal of Eating Disorders*, *40*(6), 531-536. | 33 treatment-seeking Canadian women with “prominent bulimic symptoms.” Mean age = 23.7. Mean BMI = 22.02. Race/ethnicity not reported. | NA measured using 5 items rated using a Likert scale (worried/anxious, frustrated, angry/hostile, unhappy, depressed/blue). Binge eating was defined as consumption of an objectively large amount of food over a short period of time, accompanied by LOC. | Three random times per day and before and after each eating episode for 2 weeks. | Greater NA predicted greater odds of binge eating at the next timepoint. | Low, due to insufficient discussion of low power and relatively high dropout rates (12/45 participants). |
| Fischer, S., Wonderlich, J., Breithaupt, L., Byrne, C., & Engel, S. (2018). Negative urgency and expectancies increase vulnerability to binge eating in bulimia nervosa. *Eating Disorders*, *26*(1), 39-51. | 17 women with at least one episode of binge eating and compensatory behavior in the past month. Ages 18-40 (mean age = 24.47). 71% white, 14% Latina, 14% Asian. | NA was measured using items from the PANAS and POMS (afraid, nervous, upset, hostile, ashamed, guilty, sad, tired, bored, angry). Binge eating was defined as “eating an objectively large amount of food, during a relatively short period of time, with an associated loss of control.” | Six times per day for two weeks, plus event contingent ratings made directly after eating. | NA was on an increasing trajectory prior to binge eating, and a decreasing trajectory after binge eating. Participants with lower negative urgency and lower expectancies that binge eating would help relieve negative emotions experienced a greater and more rapid increase in NA prior to binge eating, and a steeper decrease in NA after binge eating. | Medium, due to insufficient power, but adequate study measures, sampling duration/frequency, and reporting of results. |
| Fitzsimmons‐Craft, E. E., Ciao, A. C., & Accurso, E. C. (2016). A naturalistic examination of social comparisons and disordered eating thoughts, urges, and behaviors in college women. *International Journal of Eating Disorders*, *49*(2), 141-150. | 232 undergraduate women. Ages 17-22 (mean age = 18.70). Mean BMI = 22.56. 68.9% white, 7.7% Black/African American, 7.7% Asian, 4.3% Latina, 1.3% American Indian or Alaskan Native, 9.8% multiethnic, 0.4% other races/ethnicities. | NA measured using a five-item version of the NA subscale of the PANAS (distressed, upset, scared, nervous, afraid). Binge eating urges measured using the item: “have you had the urge to binge eat?”; and binge eating behavior assessed using the item: “did you actually binge eat since the last time you were signaled?” Binge eating defined as “eating an unusually large amount of food in a discrete period of time and feeling a sense of loss of control over your eating.” | Three times per day for two weeks. | Greater NA at the prior signal predicted greater binge eating urges, even after controlling for body dissatisfaction and binge eating urges at the last signal. However, NA at the prior signal was not significantly related to binge eating behavior (though the association was positive). | Medium, due to use of a convenience sample and low sampling frequency, but adequate sampling duration, reporting of results, power, and validation of study measures. |
| Fuller-Tyszkiewicz, M., Richardson, B., Skouteris, H., Austin, D., Castle, D., Busija, L.,...Broadbent, J. (2014). Optimizing prediction of binge eating episodes: A comparison approach to test alternative conceptualizations of the affect regulation model. *Journal of Eating Disorders*, 2(1), 1-8. | 93 undergraduate women ages 18-40 (mean age = 24.72). Mean BMI = 24.12. | NA was assessed using two negative mood items from the Trait Affect Scale, modified to refer to “right now.” Binge eating assessed using items from the Questionnaire for Eating and Weight Patterns – Revised assessing DSM symptoms of binge eating. | Seven times per day for one week. | A threshold of 6 or greater on an 11-point NA scale (i.e., comparing NA scores of 0-5 to 6-10) was associated with 3.68-5.58 times greater odds of binge eating. | Medium due to use of a convenience sample and somewhat brief sampling duration, but adequate power, adequate measures of NA and binge eating, and adequate reporting of results. |
| Goldschmidt, A. B., Crosby, R. D., Cao, L., Engel, S. G., Durkin, N., Beach, H. M.,...Peterson, C. B. (2014). Ecological momentary assessment of eating episodes in be obese adults. *Psychosomatic Medicine*, *76*(9), 747-752. | 50 obese adults (84% women; see Berg et al., 2015). | NA measured with a modified version of the PANAS (afraid, nervous, jittery, ashamed, disgusted, dissatisfied with self, angry with self, irritable, angry, lonely, sad). Participants rated the extent to which they felt they had overeaten (1 item) and felt LOC (4 items) for each eating episode. Binge eating defined as a score of ≥ 3 on the overeating item and at least one LOC item. | Six times per day for two weeks, plus event contingent ratings made directly after eating. | Binge eating and LOC over smaller amounts of food were associated with higher pre- and post-eating NA than normal eating episodes. Binge eating was associated with higher pre- and post-eating NA than LOC over smaller amounts of food. | High, due to well defined and validated measures, appropriate sampling frequency and duration, adequate reporting of results, and an appropriate community sample. Though power was relatively low, this was adequately acknowledged in the paper. |
| Goldschmidt, A. B., Crosby, R. D., Cao, L., Wonderlich, S. A., Mitchell, J. E., Engel, S. G., & Peterson, C. B. (2018). A preliminary study of momentary, naturalistic indicators of binge‐eating episodes in adults with obesity. *International Journal of Eating Disorders*, *51*(1), 87-91. | 50 obese adults (84% women; see Berg et al., 2015). | NA measured with a modified version of the PANAS (afraid, nervous, jittery, ashamed, disgusted, dissatisfied with self, angry with self, irritable, angry, lonely, sad). Participants rated the extent to which they felt they had overeaten (1 item) and felt LOC (4 items) for each eating episode. Binge eating defined as a score of ≥ 3 on the overeating item and at least one LOC item. | Six times per day for two weeks, plus event contingent ratings made directly after eating. | Binge eating episodes were characterized by greater likelihood of reporting moderate to high pre-eating shame and post-eating disgust depression, and/or guilt compared to non-binge eating episodes. | High, due to well defined and validated measures, appropriate sampling frequency and duration, adequate reporting of results, and an appropriate community sample. Though power was relatively low, this was adequately acknowledged in the paper. |
| Goldschmidt, A. B., Engel, S. G., Wonderlich, S. A., Crosby, R. D., Peterson, C. B., Le Grange, D.,...Mitchell, J. E. (2012). Momentary affect surrounding loss of control and overeating in obese adults with and without binge eating disorder. *Obesity*, *20*(6), 1206-1211. | 22 obese adults (86.4% women). Mean age = 35.7. Mean BMI = 38.9. 90.9% white, 4.5% Black/African American, 4.5% Native American. | NA assessed using the PANAS short form. LOC was rated prior to eating using a single Likert item (“please rate how much control you currently feel over your eating”). | Before and after every eating episode for 7 days. | Greater pre-meal NA was associated with greater (anticipated) LOC over eating. For participants with BED only, greater LOC was associated with greater post-meal NA, while for participants without BED, greater LOC and greater caloric intake were associated with lower post-meal NA. | Low, due to low power and questions about outcome measure validity (i.e., using a single item that captured anticipated LOC rather than actual LOC). |
| Goldschmidt, A. B., Peterson, C. B., Wonderlich, S. A., Crosby, R. D., Engel, S. G., Mitchell, J. E.,...Berg, K. C. (2013). Trait‐level and momentary correlates of bulimia nervosa with a history of anorexia nervosa. *International Journal of Eating Disorders*, *46*(2), 140-146. | 122 women with BN (the subset of Becker et al., 2018 with BMI ≥ 18.5). | NA measured using a modified version of the PANAS (afraid, lonely, irritable, ashamed, disgusted, nervous, dissatisfied with self, jittery, sad, angry at self, and distressed). Binge eating defined as “an amount of food that you consider excessive, or an amount of food that other people would consider excessive, with an associated loss of control or the feeling of being compelled to eat”. | Six times per day for two weeks, plus event contingent ratings made directly after eating. | Participants with and without a history of prior AN did not significantly differ in their trajectories of NA before and after binge eating. | High, due to adequate power, an appropriate (though non-fully representative) sample, good sampling frequency/duration, adequately clear and validated measures, adequate reporting of results. |
| Goldschmidt, A. B., Wonderlich, S. A., Crosby, R. D., Cao, L., Engel, S. G., Lavender, J. M.,...Le Grange, D. (2014). Latent profile analysis of eating episodes in anorexia nervosa. *Journal of Psychiatric Research*, *53*, 193-199. | 118 women with AN (see Berg et al., 2017). | NA measured using a modified version of the PANAS (nervous, disgusted, distressed, ashamed, angry at self, afraid, sad, and dissatisfied with self). Binge eating defined as “an amount of food that you consider excessive, or an amount of food that other people would consider excessive, with an associated loss of control or the feeling of being compelled to eat”. | Six times per day for two weeks, plus event contingent ratings made directly after eating. | LOC and binge eating were associated with higher levels of concurrent NA than solitary eating, restrictive eating, or avoidant eating as identified through latent profile analysis. | High, due to adequate power, an appropriate (though non-fully representative) sample, good sampling frequency/duration, adequately validated measures, adequate reporting of results. |
| Goldschmidt, A. B., Wonderlich, S. A., Crosby, R. D., Engel, S. G., Lavender, J. M., Peterson, C. B.,...Mitchell, J. E. (2014). Ecological momentary assessment of stressful events and negative affect in bulimia nervosa. *Journal of Consulting and Clinical Psychology*, *82*(1), 30-39. | 133 women with BN (see Becker et al., 2018). | NA measured using a modified version of the PANAS (afraid, lonely, irritable, ashamed, disgusted, nervous, dissatisfied with self, jittery, sad, angry at self, and distressed). Binge eating defined as “an amount of food that you consider excessive, or an amount of food that other people would consider excessive, with an associated loss of control or the feeling of being compelled to eat”. | Six times per day for two weeks, plus event contingent ratings made directly after eating. | Changes in NA from time 1 to time 2 mediated the relationship between time 1 stressors (including interpersonal stressors, general daily hassles, and stress appraisal) and time 2 binge eating. | High, due to adequate power, an appropriate (though non-fully representative) sample, good sampling frequency/duration, adequately clear and validated measures, adequate reporting of results. |
| Greeno, C. G., Wing, R. R., & Shiffman, S. (2000). Binge antecedents in obese women with and without binge eating disorder. *Journal of Consulting and Clinical Psychology*, *68*(1), 95-102. | 79 women ages 18-55 (mean age = 39) with BMI between 30 and 45 presenting for weight loss treatment. Participants could have no significant physical or psychiatric disorders, including current depression. Race/ethnicity not reported. | NA was assessed with an item that asked the extent to which a participant felt miserable, and an overall mood item rated from “very poor” to “very good”. Binge eating was rated with a single dimensional item (rated “yes” to “no” with varying degrees of intensity). The exact definition of binge eating was not provided. | Before and after each eating episode, and every 1-2 hours outside of eating, for 6 days. | In women who met criteria for BED, binge eating episodes were associated with worse mood. However, in women without BED, binge eating and mood were unrelated. | Medium, due to a somewhat unclear definition of LOC eating, exclusion criteria that may limit generalizability, and some reporting limitations (e.g., no confidence intervals), but adequate sampling duration and power. |
| Heron, K. E., Scott, S. B., Sliwinski, M. J., & Smyth, J. M. (2014). Eating behaviors and negative affect in college women's everyday lives. *International Journal of Eating Disorders*, *47*(8), 853-859. | 127 undergraduate women with no current eating disorder diagnosis, but elevated scores on the EDE-Q and Body Shape Questionnaire. Mean age = 19.6. Mean BMI = 25.5. 71% white, 18% Asian, 11% other ethnicity. | NA was assessed with 5 items from the PANAS-X selected to include high and low arousal states (angry, worried, sad, unhappy, frustrated). LOC eating was measured using an item adapted from the EDE-Q – “Did you lose control over your eating?” – which was clarified to mean “could not stop eating, even if you wanted to.” | Five times per day for one week. | There was no significant association between T1 NA and LOC eating at the next signal, after controlling for T1 LOC eating. However, T1 LOC was associated with greater NA at the next signal. | Medium, due to a sample that may not be fully generalizable to people with clinically significant LOC eating and a relatively short assessment period, but adequately validated measures, adequate statistical analyses, and adequate reporting. |
| Johnson, C., & Larson, R. (1982). Bulimia: An analysis of moods and behavior. *Psychosomatic Medicine*, *44*(4), 341–351. | Women with BN (*n* = 15) and women without an ED (*n* = 24). Ages 19-32 (mean = 23 for BN group, 24 for control group). Race/ethnicity not reported. | NA assessed using 3 dimensional items: cheerful/irritable, happy/sad, excited/bored, as well as an additional item assessing guilt. LOC eating not clearly defined. | Every two hours for one week. | Participants with BN reported feeling more irritable than average before binge eating. After binge eating or purging, participants reported feeling significantly sadder and guiltier than average. The small number of participants who received a signal while they were binge eating reported feeling guilty and inadequate at that time. | Low, due to low power and an unclearly defined measure of LOC eating. |
| Kornacka, M., Czepczor-Bernat, K., Napieralski, P., & Brytek-Matera, A. (2020). Rumination, mood, and maladaptive eating behaviors in overweight and healthy populations. *Eating and Weight Disorders-Studies on Anorexia, Bulimia and Obesity*, 1-13. | 26 individuals (11 normal weight, 15 overweight; mean age in the mid-20s) recruited through an online Facebook advertisement. Participants required to have no ED diagnosis. | NA was rated with two bipolar items: content-discontent and well-unwell. Emotional eating was assessed with three items adapted from the Three-Factor Eating Questionnaire with respect to the last time the person ate. | Five times per day for 5 days. | The more a participant reported eating in response to emotions when they last ate, the more negative their mood at the time of the signal. When rumination was added to the model, mood was no longer significantly associated with emotional eating. | Low, due to low power and a non-representative sample with potentially limited generalizability to the overall population with LOC eating. |
| Lavender, J. M., De Young, K. P., Wonderlich, S. A., Crosby, R. D., Engel, S. G., Mitchell, J. E.,...Le Grange, D. (2013). Daily patterns of anxiety in anorexia nervosa: Associations with eating disorder behaviors in the natural environment. *Journal of Abnormal Psychology*, *122*(3), 672-683. | 118 women with AN (see Berg et al., 2017). | NA was measured using 8 items from the tension-anxiety scale of the POMS (on edge, restless, tense, anxious, uneasy, shaky, panicky, relaxed [reverse coded]). Binge eating was defined as eating “an amount of food that most people would consider excessive” along with the inability to stop eating or prevent the eating episode. | Six times per day for two weeks, plus event contingent ratings made directly after eating. | Rates of binge eating were significantly higher on days characterized by stable high, late increasing, and late decreasing anxiety, relative to days characterized by stable low anxiety. Binge eating was most likely to occur in the evening on stable low anxiety days and late increasing anxiety days, in the afternoon on stable high and late decreasing anxiety days, and equivalently throughout the day on early increasing and early decreasing anxiety days. | High, due to adequate power, an appropriate (though non-fully representative) sample, good sampling frequency/duration, adequately validated measures, adequate reporting of results. |
| Lavender, J. M., Utzinger, L. M., Cao, L., Wonderlich, S. A., Engel, S. G., Mitchell, J. E., & Crosby, R. D. (2016). Reciprocal associations between negative affect, binge eating, and purging in the natural environment in women with bulimia nervosa. *Journal of Abnormal Psychology*, *125*(3), 381-386. | 133 women with BN (see Becker et al., 2018). | NA measured using a modified version of the PANAS (afraid, lonely, irritable, ashamed, disgusted, nervous, dissatisfied with self, jittery, sad, angry at self, and distressed). Binge eating defined as “an amount of food that you consider excessive, or an amount of food that other people would consider excessive, with an associated loss of control or the feeling of being compelled to eat”. | Six times per day for two weeks, plus event contingent ratings made directly after eating. | Greater NA was associated with greater likelihood of binge eating within 30 minutes of the signal. Later in the day, NA was prospectively associated with greater likelihood of binge eating at the next signal (approximately 2.5 hours later). Binge eating was generally associated with lower NA at the next signal (again, approximately 2.5 hours later), controlling for NA at approximately the time of binge eating. | High, due to adequate power, an appropriate (though non-fully representative) sample, good sampling frequency/duration, adequately clear and validated measures, adequate reporting of results. |
| Le Grange, Gorin, A., Catley, D., & Stone, A. A. (2001). Does momentary assessment detect binge eating in overweight women that is denied at interview? *European Eating Disorders Review*, *9*(5), 309-324. | Overweight women with (*n* = 18) or without (*n* = 17) BED. Participants with a current mood disorder or antidepressant medication in the past 6 months were excluded. Mean age = 45.57. Mean BMI = 35.90. >94% white. | NA rated with a single dimensional item at random prompts (exact wording not indicated), and additionally with a rating of guilt after binge eating. Binge eating defined as eating what most people would consider an unusually large amount of food in about 2 hours, accompanied by loss of control. | 5-6 times per day for two weeks, plus immediately after binge eating. | Participants with and without BED reported greater NA immediately after binge eating than at other times of day. | Low, due to low power, an unclearly operationalized measure of NA, and a sample that may not fully generalize to women with binge eating due to exclusion criteria. |
| Lingswiler, V. M., Crowther, J. H., & Stephens, M. A. (1989a). Affective and cognitive antecedents to eating episodes in bulimia and binge eating. *International Journal of Eating Disorders*, *8*(5), 533-539. | Women with BN (*n* = 19), binge eating without compensation (*n* = 15), or no eating disorder (*n* = 20). Mean age = 18.5 for the BN group, 18.4 for the binge eating group, and 19.9 for the control group. Race/ethnicity not reported. | NA rated with a single mood item ranging from “very negative” to “very positive”. LOC eating defined as a period of uncontrolled, excessive eating. | After every eating episode for one week. Participants were instructed to imagine how they had felt prior to eating. | Participants with BN and binge eating recalled experiencing significantly greater NA prior to non-LOC eating episodes than control participants did prior to eating. NA did not differ between LOC eating and non-LOC eating episodes in participants with BN and binge eating. | Low, due to low power, lack of validation of the LOC and NA measures, and no reporting of effect sizes or confidence intervals. |
| Lingswiler, V. M., Crowther, J. H., & Stephens, M. A. P. (1989b). Emotional and somatic consequences of binge episodes. *Addictive Behaviors*, *14*(5), 503-511. | Women with BN (*n* = 19), binge eating without compensation (*n* = 15), or no eating disorder (*n* = 20) (see Lingswiler et al., 1989a). | NA measured using a 13-item composite (angry at myself, anguish, anxiety, depressed, disgusting, fat/overweight, fearful, guilty, hate myself, helpless, shameful, stressed, tired/fatigued). LOC eating defined as a period of uncontrolled, excessive eating. | Immediately and one hour after every eating episode for one week. | Participants with BN and binge eating reported significantly greater NA immediately and one hour after LOC eating episodes than control participants reported after normal eating. Additionally, participants with BN reported greater NA after non-LOC eating episodes than control participants reported after normal eating. In both participants with BN and binge eating, NA was greater immediately and one hour after LOC eating episodes than after non-LOC eating episodes. | Low, due to low power and lack of validation of the LOC and NA measures. |
| Mason, T. B., Lavender, J. M., Wonderlich, S. A., Crosby, R. D., Engel, S. G., Mitchell, J. E.,...Peterson, C. B. (2018). Examining a momentary mediation model of appearance-related stress, anxiety, and eating disorder behaviors in adult anorexia nervosa. *Eating and Weight Disorders-Studies on Anorexia, Bulimia and Obesity*, *23*(5), 637-644. | 118 women with AN (see Berg et al., 2017). | Momentary anxiety measured using 8 items from the tension-anxiety scale of the POMS. Binge eating defined as “an amount of food that you consider excessive, or an amount of food that other people would consider excessive, with an associated loss of control or the feeling of being compelled to eat”. | Six times per day for two weeks, plus event contingent ratings made directly after eating. | Momentary anxiety appeared to fully mediate the association between appearance-related stress at time 1 and binge eating at time 2. | High, due to adequate power, an appropriate (though non-fully representative) sample, good sampling frequency/duration, adequately validated measures, adequate reporting of results. |
| Mason, T. B., Lavender, J. M., Wonderlich, S. A., Steiger, H., Cao, L., Engel, S. G.,...Crosby, R. D. (2017). Comfortably numb: The role of momentary dissociation in the experience of negative affect around binge eating. *The Journal of Nervous and Mental Disease*, *205*(5), 335-339. | 133 women with BN (see Becker et al., 2018). | NA measured using a modified version of the PANAS (afraid, lonely, irritable, ashamed, disgusted, nervous, dissatisfied with self, jittery, sad, angry at self, and distressed). Binge eating defined as “an amount of food that you consider excessive, or an amount of food that other people would consider excessive, with an associated loss of control or the feeling of being compelled to eat”. | Six times per day for two weeks, plus event contingent ratings made directly after eating. | NA increased more rapidly prior to binge eating, and decreased more rapidly following binge eating, when participants reported greater dissociation relative to their average. Individuals who were generally higher in dissociative tendencies experienced greater NA at the time of binge eating, and a more rapid decrease in NA following binge eating. | High, due to adequate power, an appropriate (though non-fully representative) sample, good sampling frequency/duration, adequately clear and validated measures, adequate reporting of results. |
| Moskovich, A. A., Dmitrieva, N. O., Babyak, M. A., Smith, P. J., Honeycutt, L. K., Mooney, J., & Merwin, R. M. (2019). Real-time predictors and consequences of binge eating among adults with type 1 diabetes. *Journal of Eating Disorders*, *7*(1), 7. | 83 adults with type I diabetes (88% women). Ages 18-68 (mean = 41.9). 87% white. | NA was rated using five emotion items (sad, frustrated, angry, anxious or nervous, guilty or disgusted with yourself). Binge eating was assessed by first asking participants if they ate a large amount of food (yes/no), then the extent to which they felt a loss of control over eating (rated on a 1-6 scale). | 1-2 times per hour for three days. | At a between-person level, participants who had higher average levels of NA experienced more binge eating. NA increased more following binge eating episodes than following other kinds of eating episodes, with particularly large effects for “frustrated” and “guilty or disgusted with yourself” when emotions were examined individually. | High, due to adequate power, an appropriate and generally representative sample, good sampling frequency, clear and appropriate measures, adequate reporting of results. |
| Munsch, S., Meyer, A. H., Quartier, V., & Wilhelm, F. H. (2012). Binge eating in binge eating disorder: A breakdown of emotion regulatory process? *Psychiatry Research*, *195*(3), 118-124. | 22 German women with BED participating in a CBT treatment trial. Ages 21-65 (mean age = 45.5). Mean BMI = 33.4. 27% with a mood or anxiety disorder. | NA measured using scales of negative mood (discontented, depressed, queasy, bored, anxious, lonely, sad) and tension (calm, nervous, agitated, stressed out, tense, annoyed) derived through factor analysis. Binge eating defined as eating an unusually large amount of food with loss of control. | Five times per day for one week, and whenever binge eating occurred. | Negative mood (but not tension) was greater on days when participants experienced binge eating than on days when they did not. Negative mood and tension underwent a curvilinear increase prior to the first binge eating episode, and began to decrease in a linear fashion following binge eating. When affect ratings 30 minutes before and after binge eating were removed, negative mood and tension decreased somewhat prior to binge eating, were greater 30 minutes after binge eating than 30 minutes before binge eating, and decreased from 30 minutes after binge eating on. | Medium, due to small sample size, but adequately clear and validated study measures, an adequate sampling period, reporting of effect sizes (but not CIs), and a sample that was appropriate but potentially not fully generalizable. |
| Pearson, C. M., Mason, T. B., Cao, L., Goldschmidt, A. B., Lavender, J. M., Crosby, R. D.,...Peterson, C. B. (2018). A test of a state-based, self-control theory of binge eating in adults with obesity. *Eating Disorders*, *26*(1), 26-38. | 50 obese adults (84% women; see Berg et al., 2015). | NA measured with a modified version of the PANAS (afraid, nervous, jittery, ashamed, disgusted, dissatisfied with self, angry with self, irritable, angry, lonely, sad). Participants rated the extent to which they felt they had overeaten (1 item) and felt LOC (4 items) for each eating episode. Binge eating defined as a score of ≥ 3 on the overeating item and at least one LOC item. | Six times per day for two weeks, plus event contingent ratings made directly after eating. | There was a significant two-way interaction between NA and eating expectancies, and a significant three-way interaction between NA, eating expectancies, and dietary restraint, suggesting that binge eating was most likely when NA, the expectation that eating would help one feel better, and dietary restraint were all higher than typical for an individual. | Medium, due to relatively low power that was not adequately acknowledged, but well defined and validated measures, appropriate sampling frequency and duration, adequate reporting of results, and an appropriate community sample. |
| Pollert, G. A., Engel, S. G., Schreiber‐Gregory, D. N., Crosby, R. D., Cao, L., Wonderlich, S. A.,...Mitchell, J. E. (2013). The role of eating and emotion in binge eating disorder and loss of control eating. *International Journal of Eating Disorders*, *46*(3), 233-238. | Obese adults with BED (*n* = 9) or no BED (*n* = 13). Gender not reported. Participants with a “current psychological disorder” were excluded. | NA was assessed using five items from the PANAS with the highest factor loadings (specific items not listed). LOC assessed after every eating episode with a single item asking whether the participant experienced LOC (yes/no). LOC was defined to participants as ‘‘feeling ‘driven’ or ‘compelled’ to eat,’’ feeling ‘‘unable to stop eating once eating had started,’’ or feeling ‘‘unable to prevent the episode from occurring.’’ | Six times per day for one week, and before and after eating. | Greater NA was associated with greater LOC over eating after controlling for BED group membership and the number of calories consumed. However, the results do not make clear whether this was NA measured before or after eating. | Low, due to low power, unclear reporting, and limitations to generalizability given exclusion of participants with comorbid mental health conditions. |
| Rebert, W. M., Stanton, A. L., & Schwarz, R. M. (1991). Influence of personality attributes and daily moods on bulimic eating patterns. *Addictive Behaviors*, *16*(6), 497-505. | Undergraduate women with BN (*n* = 13) and binge eating without compensatory behaviors (*n* = 13). | NA measured using the depression, anxiety, and hostility subscales of the Multiple Affect Adjective Checklist and the State-Trait Anxiety Inventory state form. Binge eating defined subjectively by participants based on their perception of a binge; only eating episodes of at least 1,000 calories were included in analyses. | Twice per day for 20 days. | Greater depression, anxiety, and hostility earlier in the day predicted greater likelihood of subsequent binge eating. This association was of similar magnitude for participants with BN and regular binge eating. Individuals with greater trait hostility and an external locus of control were more likely to binge following negative moods. | Low, due to low power and an unclearly defined measure of LOC eating. |
| Redlin, J. A., Miltenberger, R. G., Crosby, R. D., Wolff, G. E., & Stickney, M. I. (2002). Functional assessment of binge eating in a clinical sample of obese binge eaters. *Eating and Weight Disorders-Studies on Anorexia, Bulimia and Obesity*, *7*(2), 106-115. | 10 treatment-seeking women with BED. Mean age = 44.7. Mean BMI = 42.4. Race/ethnicity not reported. | NA measured using 13 emotion items (anxiety, boredom, anger at self, anger at others, dissatisfaction, worry-responsibility, worry-problems, worry-future, loneliness, frustration, sadness, guilt, agitation). Binge eating defined as consumption of a large amount of food over a short period of time, accompanied by loss of control. | Before, immediately after, and slightly longer after LOC eating for 1-5 weeks (reasons for differences in sampling duration not stated). | Anxiety, boredom, anger at others, worry about responsibility, worry about problems, loneliness, frustration, and agitation were lower after binge eating than before binge eating. Anger at self, dissatisfaction, worry about the future, sadness, and guilt were greater after binge eating than before binge eating. However, it is unclear whether any of these differences were statistically significant. | Low, due to low power and lack of inferential statistics. |
| Sanftner, J. L., & Crowther, J. H. (1998). Variability in self‐esteem, moods, shame, and guilt in women who binge. *International Journal of Eating Disorders*, *23*(4), 391-397. | Undergraduate women with (*n* = 37) and without (*n* = 41) binge eating. Participants could not have a history of AN or BN. Mean age = 19.4. Mean BMI = 24.8 for the binge eating group and 22.8 for the control group. Race/ethnicity not reported. | NA assessed using the PANAS and the State Self-Esteem Scale (which captures guilt/shame). LOC eating not clearly defined. | Every four hours for one week. | Participants with binge eating reported greater guilt/shame than participants without binge eating, but the group difference did not significantly change around binge eating episodes. NA did not significantly change from nine hours before binge eating to one hour before binge eating. | Low, due to relatively low power, an unclearly defined measure of binge eating, and lack of appropriate statistical analyses (i.e., accounting for repeated measures). |
| Schaefer, L. M., Smith, K. E., Anderson, L. M., Cao, L., Crosby, R. D., Engel, S. G.,...Wonderlich, S. A. (2020). The role of affect in the maintenance of binge-eating disorder: Evidence from an ecological momentary assessment study. *Journal of Abnormal Psychology*, *129*(4), 387-396. | 112 adults with BED in a treatment trial (82.1% women). Ages 18-64 (mean age = 39.7). Mean BMI = 35.1. 91.1% white. 57.1% with lifetime mood disorders, 37.5% with lifetime anxiety disorders. | NA measured using 16 items from the PANAS-X (afraid, nervous, scared, frightened, upset, ashamed, disgusted with self, dissatisfied with self, angry at self, scornful, disgusted, hostile, disdainful, lonely, sad, alone). Binge eating was assessed through a series of questions asking about perceived overeating (“To what extent do you feel that you overate?”, “To what extent do you feel that you ate an excessive amount of food?”) and LOC (“While you were eating, to what extent did you feel a sense of loss of control?”, “While you were eating, to what extent did you feel that you could not resist eating?”, “While you were eating, to what extent did you feel that you could not stop eating once you had started?”, “While you were eating, to what extent did you feel driven or compelled to eat?”). An episode was characterized as binge eating if it was rated 4 or higher on a 1-5 scale for at least one overeating item and at least one LOC item. | Five times per day for one week. | NA increased in a curvilinear fashion prior to binge eating, and began to decrease in a linear fashion following binge eating. With respect to specific facets of NA, emotions related to fear, sadness, and hostility remained relatively stable across binge eating episodes. However, guilt increased in a curvilinear manner prior to binge eating and decreased in a linear fashion after binge eating. | High, due to adequate power, an appropriate (though non-fully representative) sample, good sampling frequency/duration, adequately clear and validated measures, adequate reporting of results. |
| Selby, E. A., Doyle, P., Crosby, R. D., Wonderlich, S. A., Engel, S. G., Mitchell, J. D., & Le Grange, D. (2012). Momentary emotion surrounding bulimic behaviors in women with bulimia nervosa and borderline personality disorder. *Journal of Psychiatric Research*, *46*(11), 1492-1500. | 133 women with BN (see Becker et al., 2018). | NA measured using a modified version of the PANAS (afraid, lonely, irritable, ashamed, disgusted, nervous, dissatisfied with self, jittery, sad, angry at self, and distressed). Binge eating defined as “an amount of food that you consider excessive, or an amount of food that other people would consider excessive, with an associated loss of control or the feeling of being compelled to eat”. | Six times per day for two weeks, plus event contingent ratings made directly after eating. | NA was on an increasing trajectory prior to binge eating, and a decreasing trajectory following binge eating. Similar patterns were seen in women with BN only and women with BN and comorbid borderline personality disorder. | High, due to adequate power, an appropriate (though non-fully representative) sample, good sampling frequency/duration, adequately clear and validated measures, adequate reporting of results. |
| Sherwood, N. E., Crowther, J. H., Wills, L., & Ben-Porath, Y. S. (2000). The perceived function of eating for bulimic, subclinical bulimic, and non-eating disordered women. *Behavior Therapy*, *31*(4), 777-793. | Undergraduate women with BN (*n* = 20), subthreshold BN (*n* = 32), or no eating disorder (*n* = 28). Mean age = 19.1 for the BN group, 19.4 for the subthreshold BN group, and 18.5 for the control group. Mean BMI = 25.1 for the BN group, 25.7 for the subthreshold BN group, and 23.4 for the control group. Race/ethnicity not reported. | NA measured using the PANAS. LOC eating not clearly defined. | Before, immediately after, and one hour after eating for one week. | In women with BN and subthreshold BN, NA increased significantly from before to immediately after binge eating, and from immediately after binge eating to an hour after binge eating. Conversely, NA decreased from before to after normal eating episodes. The magnitude of these effects did not differ between BN and subthreshold BN groups. | Low, due to an unclear definition of LOC eating, and relatively low power without acknowledgement of this limitation. |
| Smith, K. E., Mason, T. B., Crosby, R. D., Engel, S. G., Crow, S. J., Wonderlich, S. A., & Peterson, C. B. (2018). State and trait positive and negative affectivity in relation to restraint intention and binge eating among adults with obesity. *Appetite*, *120*, 327-334. | 50 obese adults (84% women; see Berg et al., 2015). | NA measured with a modified version of the PANAS (afraid, nervous, jittery, ashamed, disgusted, dissatisfied with self, angry with self, irritable, angry, lonely, sad). Participants rated the extent to which they felt they had overeaten (1 item) and felt LOC (4 items) for each eating episode. Binge eating defined as a score of ≥ 3 on the overeating item and at least one LOC item. | Six times per day for two weeks, plus event contingent ratings made directly after eating. | Individuals with greater average levels of NA reported more binge eating. Higher momentary NA predicted a greater likelihood of subsequent binge eating. | High, due to well defined and validated measures, appropriate sampling frequency and duration, adequate reporting of results, and an appropriate community sample. Though power was somewhat low, this was adequately acknowledged in the paper. |
| Smith, K. E., Mason, T. B., Crosby, R. D., Engel, S. G., & Wonderlich, S. A. (2019). A multimodal, naturalistic investigation of relationships between behavioral impulsivity, affect, and binge eating. *Appetite*, *136*, 50-57. | 30 women with regular binge eating. Mean age = 36.07. Mean BMI = 34.73. 93% white. | NA measured using the PANAS short form. Binge eating rated yes/no on EMA, but not clearly defined. | Five times per day for two weeks. | Momentary NA was associated with greater likelihood of binge eating reported at the next signal, but only for people with greater delay discounting (preference for smaller but immediate rewards). | Low, due to low power and an unclearly defined measure of LOC eating. |
| Smith, K. E., Mason, T. B., Juarascio, A., Weinbach, N., Dvorak, R., Crosby, R. D., & Wonderlich, S. A. (2020a). The momentary interplay of affect, attention bias, and expectancies as predictors of binge eating in the natural environment. *International Journal of Eating Disorders*, *53*(4), 586-594. | Women with BED (*n* = 29), BN (*n* = 9), AN-BP (*n* = 1), and subthreshold BED (*n* = 1). Ages 19-64 (mean age = 34.70). Mean BMI = 34.30. 87.5% white. | NA measured using the PANAS short form, plus an item measuring guilt. Binge eating was assessed with items measuring overeating (“To what extent do you: feel that you overate?; think that others would consider what you ate to be an usual or excessive amount of food?”) and LOC (“While you were eating, to what extent did you: feel a sense of loss of control?; feel that you could not stop eating once you started?; feel disconnected [e.g., numb, zoned out, on auto-pilot]?”). An eating episode was categorized as a binge if the average scores on the overeating and LOC items were both ≥ 3. | Five times per day for 10 days, and after eating episodes. | Higher within-person NA and attentional bias toward food cues were related to increased likelihood of binge eating at the next signal. There was also a significant two-way interaction between within-person NA and within-person eating expectancies, such that the association between within-person NA and binge eating was greater during moments when people more strongly expected that food would help them feel better. | Medium, with relatively low power that was not acknowledged, but clear and adequately validated measures of NA and binge eating, an appropriate sample, an adequate sampling frequency and duration, and adequate reporting of results. |
| Smith, K. E., Mason, T. B., Schaefer, L. M., Anderson, L. M., Hazzard, V. M., Crosby, R. D.,...Peterson, C. B. (2020b). Micro-level de-coupling of negative affect and binge eating in relationship to macro-level outcomes in binge eating disorder treatment. *Psychological Medicine*, 1-9. | 78 adults with BED in a treatment trial (86.4% women; a subsample of Schaefer et al., 2020). Ages 18-64 (mean age = 40.01). Mean BMI = 34.14. 92.6% white, 1.2% Latinx, 1.2% Asian, 4.9% other race/ethnicity. | NA was measured using the PANAS short form. Binge eating was measured dimensionally by summing Likert ratings on items assessing overeating (“To what extent to do you feel that you overate?”, “To what extent do you feel that you ate an excessive amount of food?”) and LOC (“While you were eating, to what extent did you feel a sense of loss of control?”, “While you were eating, to what extent did you feel that you could not resist eating?”, “While you were eating, to what extent did you feel that you could not stop eating once you had started?”, “While you were eating, to what extent did you feel driven or compelled to eat?”). | Five times per day for one week, both before and after treatment. | Greater NA was associated with more binge eating symptoms at the next signal. This association was strengthened slightly at the end of treatment (across treatment groups), but weakened at 6 month follow-up. | High, due to adequate power, an appropriate (though likely non-fully representative) sample, good sampling frequency/duration, adequately clear and validated measures, adequate reporting of results. |
| Smith, K. E., Mason, T. B., Schaefer, L. M., Juarascio, A., Dvorak, R., Weinbach, N.,...Wonderlich, S. A. (2020c). Examining intra-individual variability in food-related inhibitory control and negative affect as predictors of binge eating using ecological momentary assessment. *Journal of Psychiatric Research*, *120*, 137-143. | Women with BED (*n* = 29), BN (*n* = 9), AN-BP (*n* = 1), and subthreshold BED (*n* = 1) (see Smith et al., 2020a). | NA measured using the PANAS short form, plus an item measuring guilt. Binge eating was assessed with items measuring overeating (“To what extent do you: feel that you overate?; think that others would consider what you ate to be an usual or excessive amount of food?”) and LOC (“While you were eating, to what extent did you: feel a sense of loss of control?; feel that you could not stop eating once you started?; feel disconnected [e.g., numb, zoned out, on auto-pilot]?”). An eating episode was categorized as a binge if the average scores on the overeating and LOC items were both ≥ 3. | Five times per day for 10 days, and after eating episodes. | There was no significant interaction between within person NA and responses on an ambulatory go/no-go task in predicting binge eating in the overall sample. However, in exploratory analyses, the interaction between within person NA and responses on the go/no-go task was significant in participants with compensatory behaviors only. Specifically, the association between momentary NA and binge eating was stronger on days when performance on the go/no-go task was poorer in this subsample. | High, with clear and adequately validated measures of NA and binge eating, an appropriate sample, an adequate sampling frequency and duration, and adequate reporting of results. Though power was relatively low, this was adequately acknowledged, particularly when discussing possible differences across the presence/absence of compensatory behaviors. |
| Smyth, J. M., Wonderlich, S. A., Heron, K. E., Sliwinski, M. J., Crosby, R. D., Mitchell, J. E., & Engel, S. G. (2007). Daily and momentary mood and stress are associated with binge eating and vomiting in bulimia nervosa patients in the natural environment. *Journal of Consulting and Clinical Psychology*, *75*(4), 629-638. | 131 women with BN (sample overlaps with Becker et al., 2018). Ages 18-55 (mean age = 25.3). Mean BMI = 23.2. 96.9% white. 87% with lifetime mood disorder, 59.5% with lifetime anxiety disorder. | NA measured using a modified version of the PANAS (afraid, lonely, irritable, ashamed, disgusted, nervous, dissatisfied with self, jittery, sad, angry at self, and distressed). Binge eating defined as “an amount of food that you consider excessive, or an amount of food that other people would consider excessive, with an associated loss of control or the feeling of being compelled to eat”. | Six times per day for two weeks, plus event contingent ratings made directly after eating. | Prior to binge eating, women reported increasing trajectories of NA and anger/hostility. Following binge eating, NA and anger/hostility began to decrease. Results were unchanged when observations within 10 min of the binge (i.e., potentially attributable to the binge itself) were removed. | High, due to adequate power, an appropriate (though non-fully representative) sample, good sampling frequency/duration, adequately clear and validated measures, adequate reporting of results. |
| Steiger, H., Gauvin, L., Engelberg, M. J., Kin, N. N. Y., Israel, M., Wonderlich, S. A., & Richardson, J. (2005). Mood-and restraint-based antecedents to binge episodes in bulimia nervosa: Possible influences of the serotonin system. *Psychological Medicine*, *35*(11), 1553-1562. | 21 women with BN. Mean age = 24.1. Mean BMI = 22.2. Race/ethnicity not reported. | NA was measured using five affect words from the circumplex model of emotion (worried, frustrated, angry, unhappy, depressed) summed to create a composite. Binge eating was recorded “according to written definitions and illustrative examples”; however, the exact definition was not provided in the paper. | Three times per day for two weeks. | Mood was worse prior to binge eating than on days when binge eating did not occur, and became worse after binge eating. Women with lower paroxetine-binding density showed more negative mood before and after binge eating relative to other women. | Low, due to low power, a relatively low sampling frequency, and an unclearly defined measure of LOC eating. |
| Steiger, H., Gauvin, L., Jabalpurwala, S., Séguin, J. R., & Stotland, S. (1999). Hypersensitivity to social interactions in bulimic syndromes: Relationship to binge eating. *Journal of Consulting and Clinical Psychology*, *67*(5), 765-775. | Women with BN (*n* = 43), subthreshold BN (*n* = 4), BED (*n* = 4), or AN-BP (*n* = 4); women in recovery from BN (*n* = 24); and healthy controls (*n* = 31). Mean age = 27.46 for the current ED group, 27.39 for the recovered ED group, 25.84 for the control group. Mean BMI = 21.64 for the current ED group, 22.16 for the recovered ED group, 21.81 for the control group. Race/ethnicity not reported. | NA was measured using a single mood composite with both negative and positive emotion items (annoyed, frustrated, sad, depressed, pleased, happy, satisfied, content). Principal-components analysis showed that these emotions formed a unitary measure. Binge eating was defined “according to objective definitions” provided to participants; however, the definition was not included in the paper. | After any social interaction lasting ≥ 10 minutes for 6-22 days. | After controlling for the tone of social interactions, mood prior to binge eating did not significantly differ from mood on non-binge eating days, but mood after binge eating was significantly worse than on non-binge eating days. (Effects were similar if social interaction tone was not included in the model.) | Low, due to relatively low power that was not addressed and an unclearly defined measure of LOC eating. |
| Stein, R. I., Kenardy, J., Wiseman, C. V., Dounchis, J. Z., Arnow, B. A., & Wilfley, D. E. (2007). What's driving the binge in binge eating disorder? A prospective examination of precursors and consequences. *International Journal of Eating Disorders*, *40*(3), 195-203. | 33 women with BED in a psychotherapy treatment study. Ages 28-63 (mean age = 45.2). Mean BMI = 37.3. 97% white. | NA was assessed with visual analogue scales rating mood from “extremely unpleasant” to “extremely pleasant”, and guilt rated from “not guilty” to “extremely guilty”. Participants were also prompted to report why they thought they had experienced binge eating (i.e., if this was due to how they felt). Binge eating was assessed with a question asking if binge eating had occurred, followed by a visual analogue scale rating LOC during eating from “not at all” to “totally”. | Six times per day for one week. | Mood and guilt before binge eating were similar to mood during non-binge eating times. However, mood after binge eating was significantly worse than mood during non-binge eating times, and guilt was significantly greater. 47.7% of binge eating episodes were attributed to how the participant felt. However, participants only reported stopping binge eating due to a change in how they felt 5.5% of the time. | Medium, due to low power (which was acknowledged) and somewhat limited validation of NA and binge eating measures, but an appropriate (though non-fully representative) sample, good sampling frequency/duration, adequately clear measures, adequate reporting of results. |
| Stevenson, B. L., Dvorak, R. D., Wonderlich, S. A., Crosby, R. D., & Gordon, K. H. (2018). Emotions before and after loss of control eating. *Eating Disorders*, *26*(6), 505-522. | 45 adults (73% women) with distressing LOC eating at least once per week. Ages 18-41 (mean age = 23.73). Mean BMI = 30.91. 91.5% white. | NA assessed using 15 items from the PANAS-X and mood circumplex (stress: stressed, overwhelmed, frazzled; anxious: nervous, jittery, anxious; guilt: angry with yourself, disappointed in yourself, ashamed; anger: angry, frustrated, tense; sadness: sad, downhearted, depressed). LOC eating was assessed using the item “Did you have a sense of having lost control over your eating (at the time that you were eating)?” adapted from the EDE-Q. | Nine times per day for two weeks. | Anger, anxiety, sadness, and stress were higher at the estimated point of LOC on LOC days than on non-LOC days, but trajectories of these emotions did not differ between LOC and non-LOC days. Guilt was also higher at the estimated point of LOC on LOC days. There was a greater increase in guilt prior to LOC eating relative to the same time period on non-LOC days, but no difference in guilt trajectory following the LOC time. | High, due to a power analysis demonstrating adequate power despite the relatively small sample size (likely due to the high sampling frequency), adequately validated measures of NA and LOC, good use of non-LOC eating days as a control, and an appropriate community sample. |
| Svaldi, J., Werle, D., Naumann, E., Eichler, E., & Berking, M. (2019). Prospective associations of negative mood and emotion regulation in the occurrence of binge eating in binge eating disorder. *Journal of Psychiatric Research*, *115*, 61-68. | 79 people with BED enrolled in a treatment trial (81.0% women). Mean age = 44.77. Mean BMI = 34.41. Race/ethnicity not reported. | NA was assessed with a single item indicating the extent to which they were experiencing overall negative mood on a 0-4 scale. Binge eating was assessed with yes/no questions regarding experiencing a loss of control, eating an unusually large amount in a distinct period of time, and other behaviors typically associated with binge eating based on items from the EDE (e.g., eating more rapidly than normal). | Three times per day for two weeks. | NA was generally higher on days characterized by binge eating than on non-binge eating days. NA did not significantly change from before to after binge eating. There were also no significant changes in the trajectory of NA before or after binge eating. | Medium, due to somewhat limited validity of the NA measure and low sampling frequency, but adequate sampling duration, an appropriate (though possibly not fully representative) sample, adequate power, adequate statistical analyses are reporting of results. |
| Wegner, K. E., Smyth, J. M., Crosby, R. D., Wittrock, D., Wonderlich, S. A., & Mitchell, J. E. (2002). An evaluation of the relationship between mood and binge eating in the natural environment using ecological momentary assessment. *International Journal of Eating Disorders*, *32*(3), 352-361. | 27 undergraduate women with binge eating at least once per week. Mean age = 19.4. Mean BMI = 25.0. Race/ethnicity not reported. | NA was assessed using 9 items from the POMS and PANAS (sad, hopeless, discouraged, annoyed, peeved, resentful, guilty, disgusted with self, dissatisfied with self). LOC eating not clearly defined. | Seven times per day for two weeks, and immediately after binge eating. | NA was significantly higher on days characterized by binge eating than on days without binge eating. There were no significant differences in NA between the most proximal assessments before and after binge eating. However, when participants were asked how they had felt before binge eating immediately after binge eating, they reported significantly more depression, anger, and guilt/self-blame post-binge relative to pre-binge. | Low, due to low power and an unclearly defined measure of LOC eating. |
| Williams-Kerver, G. A., Steffen, K. J., Smith, K. E., Cao, L., Crosby, R. D., & Engel, S. G. (2020). NA and loss of control eating among bariatric surgery patients: An ecological momentary assessment pilot investigation. *Obesity Surgery*, 1-6. | Adults seeking gastric bypass surgery (*n* = 14) or who had already received this surgery (*n* = 17) (90% female). Mean age = 41.16. Mean BMI for pre-surgery group = 44.70, for post-surgery group = 27.06. 95% white. | NA assessed using the PANAS short form. LOC over eating assessed with the item “Did you experience a sense of loss of control during this eating episode?” rated 1-5 when a participant reported eating since the last signal. | Six times per day for two weeks. | Greater within-person NA predicted greater LOC eating at the next signal, and the relationship between momentary NA and subsequent LOC eating was stronger among post-surgery participants and individuals who had lost a lower percentage of their body weight post-surgery. | Medium, due to relatively low power, but an appropriate (though possibly not fully representative) sample, clear and appropriate measures of NA and LOC, appropriate statistical analyses, and adequate sampling duration and frequency. |
| Wonderlich, J. A., Breithaupt, L., Thompson, J. C., Crosby, R. D., Engel, S. G., & Fischer, S. (2018). The impact of neural responses to food cues following stress on trajectories of negative and positive affect and binge eating in daily life. *Journal of Psychiatric Research*, *102*, 14-22. | 16 women with at least one episode of binge eating and compensatory behavior in the past month. Ages 18-40 (mean age = 22.85). Mean BMI = 24.47. 71.4% white, 14.3% Latina, 14.3% Asian. | NA measured using items from the PANAS (specific items not listed). LOC eating not clearly defined. | Six times per day for two weeks, plus event contingent ratings made directly after eating. | NA significantly increased prior to binge eating and decreased in the hours following binge eating. Women who showed a greater decrease in amygdala activity pre- to post-stress induction when observing food cues in a pre-EMA fMRI paradigm exhibited a greater and more rapid increase in NA prior to binge eating. Women who experienced greater decreases in vmPFC activity in response to visual food cues pre- to post-stress induction showed greater increases in NA and a more rapid increase in NA prior to binge eating, and a greater decrease in NA following binge eating. | Low, due to low power and unclearly defined measures of NA and LOC eating. |
| ***Experience Sampling Studies Assessing Affect During the LOC Episode*** | | | | | |
| **Citation** | **Sample Size and Study Demographics** | **Measures of NA and LOC Eating** | **Sampling Frequency** | **Key Findings** | **Overall Study Quality** |
| Deaver, C. M., Miltenberger, R. G., Smyth, J., Meidinger, A. M. Y., & Crosby, R. (2003). An evaluation of affect and binge eating. *Behavior Modification*, *27*(4), 578-599. | Undergraduate women with (*n* = 22) and without (*n* = 25) regular binge eating. Mean age = 19.8, mean BMI = 24.5 for the binge eating group (BMI not collected for women without binge eating). Race/ethnicity not reported. | NA measured using the pleasure-displeasure dimension of an affect grid. Binge eating indicated by participants when they felt they had eaten a large amount of food in a discrete period of time. | Participants rated their affect at 2 minute intervals before, during, and up to 30 min after eating for 4 days, or up to 7 days if two binge eating episodes had not occurred in 4 days for the binge eating group only. | Participants with binge eating reported that meals were more pleasant than binge eating episodes. Emotions were slightly more pleasant during and after binge eating than before binge eating. | Low, due to a convenience sample, low power, a measure of binge eating that did not assess the full construct (i.e., specifying LOC), and possible conflation of negative and positive affect through use of a single dimensional scale. |
| Hilbert, A., & Tuschen‐Caffier, B. (2007). Maintenance of binge eating through negative mood: A naturalistic comparison of binge eating disorder and bulimia nervosa. *International Journal of Eating Disorders*, *40*(6), 521-530. | 20 women with BED, 20 women with BN, and 20 women without an ED in Germany. Mean ages = 36.65 for BED group, 24.47 for BN group, and 36.30 for non-clinical group. Mean BMI = 32.99 for BED group, 23.13 for BN group, and 32.18 for non-clinical group. Race/ethnicity not reported. | Mood was rated with a single item from “very bad” to “very good”. Participants rated whether any eating episode was a binge (i.e., yes/no to eating a large amount of food in a discrete time, accompanied by LOC). | Mood and eating assessed 32 times per day (in 10-50 minute intervals) for 2 days. Participants also asked to rate mood before, during, and after eating. | In both BN and BED groups, mood was significantly worse prior to binge eating than prior to regular eating or at non-eating assessments. For both BN and BED groups, mood was more negative after binge eating than before or during binge eating. | Low, due to low power, limited validity for the measure of NA, short sampling duration. |
| Corstorphine, E., Waller, G., Ohanian, V., & Baker, M. (2006). Changes in internal states across the binge-vomit cycle in bulimia nervosa. *The Journal of Nervous and Mental Disease*, *194*(6), 446-449. | 23 women with DSM-IV BN recruited from a specialist ED clinic (ages 17-50, mean = 29.9), mean BMI = 24.5. Race/ethnicity not reported. | NA measured with specific emotions (anxiety/worry, anger/frustration, guilt/shame, loneliness) rated on a 0-100 scale. LOC eating not clearly defined. | Mood was assessed during every binge eating/purging episode for a week (mean 4.70 episodes, range 1-12). | Anger/frustration and loneliness were elevated before binge eating, and remained constant across the binge eating/purging cycle. Guilt/shame increased significantly after binge eating and remained elevated even after purging. Anxiety/worry increased after binge eating, then decreased after purging. | Low, due to low power and an unclearly defined measure of LOC eating. |
| Powell, A. L., & Thelen, M. H. (1996). Emotions and cognitions associated with bingeing and weight control behavior in bulimia. *Journal of Psychosomatic Research*, *40*(3), 317-328. | Women with (*n* = 22) and without (*n* = 22) BN recruited through intro psychology classes. Mean age = 20.25 for BN group, 19.33 for control group. Race/ethnicity not reported. | Items representing hostility, depression, and anxiety were chosen from the Multiple Affect Adjective Check List based on factor analytic loadings. LOC eating not clearly defined. | Mood was assessed every 2 hours and throughout the binge/purge cycle for 6 days (most participants reported 1 binge). | Composite NA was greater than typical before, during, and after binge eating, as well as after purging. NA did not significantly change from before to during or after binge eating, but did decrease after purging. Depression and hostility followed this same general pattern, while anxiety decreased to baseline levels after purging (but not directly after binge eating). | Low, due to low power and an unclearly defined measure of LOC eating. |
| Stickney, M. I., Miltenberger, R. G., & Wolff, G. (1999). A descriptive analysis of factors contributing to binge eating. *Journal of Behavior Therapy and Experimental Psychiatry*, *30*(3), 177-189. | 16 undergraduate women with binge eating at least 2x per week. Age and race/ethnicity not reported. | NA was assessed using 15 affective state items. LOC eating not clearly defined. | Participants reported mood immediately before, during, and after binge eating for 4 weeks (mean 5.88 binge eating episodes, range 0-29). | When asked to reflect on binge eating antecedents, participants reported binge eating in response to a range of negative emotions (i.e., depressed, upset, empty, hopeless, stressed, overwhelmed). Anger at self, frustration, down/sad, guilt, anxious/nervous, and agitation/irritability ratings increased from before to during/after binge eating, while boredom and loneliness decreased (abet only slightly for loneliness). | Low, due to low power, inadequate reporting of sample demographics, and an unclearly defined measure of LOC eating. |

*Note*: AN-BP = anorexia nervosa, binge eating/purging subtype; BED = binge eating disorder; BMI = body mass index; BN = bulimia nervosa; ED = eating disorder; EDE = Eating Disorder Examination; EDE-Q = Eating Disorder Examination Questionnaire; EMA = ecological momentary assessment; LOC = loss of control; NA = negative affect; PANAS = Positive and Negative Affect Schedule; POMS = Profile of Mood States.

**Appendix B.** Study quality assessment measure

| **High** | **Medium** | **Low** |
| --- | --- | --- |
| **Study Population** | | |
| The sample is representative of the overall population of people with loss of control eating. Inclusion and exclusion criteria are explicitly stated, and the authors provide evidence that these criteria do not limit the ability to generalize study findings to the broader population with loss of control eating. If the study population is restricted in some way (e.g., based on sex), a rationale is clearly stated, and the limitations for generalizability noted. | A clear rationale for the sampling method is provided; however, the sample may not be fully representative of people with loss of control eating in the general population (e.g., a treatment-seeking sample). Inclusion/exclusion criteria may limit generalizability in some important ways (e.g., excluding people with current mood disorders, which are highly comorbid with loss of control eating). | A non-clinical convenience sample is used, or the rationale for the sample is not provided. Generalizability from the sample to the broader population of individuals with loss of control eating is limited. |
| **Power** | | |
| The study is adequately powered to detect associations of at least a medium effect size. | The study is not adequately powered to detect associations of a medium effect size, but this limitation is clearly stated. | The study is not adequately powered to detect associations of a medium effect size, and this limitation is not acknowledged. Conclusions about the absence of a meaningful effect may be drawn despite lack of adequate power to detect such an effect. |
| **Statement of Study Aims/Hypotheses** | | |
| Study hypotheses are clearly stated, and have a clear rationale based on prior literature. Primary study hypotheses are differentiated from exploratory analyses. The study may have been pre-registered. | The study is primarily exploratory. The exploratory nature of analyses is clearly stated, and conclusions drawn are tentative. | Primary study hypotheses and post-hoc/exploratory analyses are not clearly differentiated. Exploratory or post-hoc analyses are discussed in definitive or confirmatory terms. |
| **Measures** | | |
| Predictor and outcome measures are clearly described and show good psychometric properties (e.g., internal consistency). The authors provide strong evidence of measure validity (e.g., associations with established measures or interviewer-assessed symptoms; discriminant validity with other constructs). Any limitations of study measures are clearly noted. | Predictor and outcome measures are clearly described and show adequate psychometric properties. At least some evidence of measure validity is provided. Any limitations of study measures are clearly noted. | Either the predictor and/or outcome measure demonstrates poor reliability or validity, or reliability or validity is not discussed. Limitations of study measures may not be acknowledged. |
| **Study Duration and Sampling Frequency** | | |
| Study duration is adequate to capture multiple instances of the behavior of interest. For EMA studies, sampling frequency is adequate to detect changes in affect that may be proximally related to loss of control eating. | Study duration is adequate to capture multiple instances of the behavior of interest, or sampling frequency is adequate to detect changes in affect that may be proximally related to loss of control eating, but one of these parameters is suboptimal. | Study duration is inadequate to capture more than one instance of the behavior for most participants, and sampling frequency in an EMA study is very low or inappropriate for the question being addressed. |
| **Statistical Modeling Approach** | | |
| The statistical modeling approach is appropriate for the question under study. Potential confounds are controlled for statistically or through the study design, and alternative explanations are ruled out. | The statistical modeling approach is appropriate for the question under study. However, some important confounds or alternative explanations may not have been addressed. | The statistical modeling approach has serious flaws that compromise study conclusions. |
| **Response Rates and Attrition** | | |
| Non-response rates and participant attrition are very low (i.e., less than 15%). | Non-response rates and participant attrition are moderate (i.e., 15-30%). The possibility of bias introduced through non-response or participant attrition is examined and adequately addressed. | Non-response rates and participant attrition are high (i.e., greater than 30%), or possible bias introduced through a moderate level of non-response or participant attrition is not adequately addressed. |
| **Reporting of Results** | | |
| Effect sizes and confidence intervals are reported for all primary study analyses. Results are clear and easy to interpret (e.g., units of measurement are clearly stated). Key demographic variables (e.g., race/ethnicity, age) are clearly reported. | Effect sizes and/or confidence intervals are reported for some results only. Demographic information is limited. | Effect sizes and confidence intervals are absent. The study may report significance values alone. Demographic information is severely limited or absent. |

*Note*: The majority of existing study quality assessment tools have focused on randomized control trials, or else lack important criteria for assessing key properties of experience sampling studies in particular (such as sampling frequency and duration). Therefore, a new study quality assessment measure was developed for this review that draws on relevant existing measures, including the Newcastle-Ottawa Quality Assessment Scale for nonrandomized studies (Wells et al., 2000) and the Cochrane Collaboration’s tool for assessing risk of study bias (Higgins et al., 2011). Overall study quality was rated “high” if a majority of domains were rated high and none were rated low, “medium” if a majority of domains were rated medium and no more than one was rated low, and “low” if two or more domains were rated low, indicating multiple serious concerns regarding study design and/or presentation of results. As shown in Appendix A, the most common reasons for a low overall study quality rating were small sample size and inadequate definition or validation of measures of negative affect and loss of control eating.
